# RPA guides UNG to uracil in ssDNA to facilitate antibody class switching and repair of mutagenic uracil at the replication fork

**DOI:** 10.1093/nar/gkad1115

**Published:** 2023-11-24

**Authors:** Abdul B Hayran, Nina B Liabakk, Per A Aas, Anna Kusnierczyk, Cathrine B Vågbø, Antonio Sarno, Tobias S Iveland, Konika Chawla, Astrid Zahn, Javier M Di Noia, Geir Slupphaug, Bodil Kavli

**Affiliations:** Department of Clinical and Molecular Medicine, NTNU Norwegian University of Science and Technology, NO-7491 Trondheim, Norway; Department of Clinical and Molecular Medicine, NTNU Norwegian University of Science and Technology, NO-7491 Trondheim, Norway; Department of Clinical and Molecular Medicine, NTNU Norwegian University of Science and Technology, NO-7491 Trondheim, Norway; Department of Clinical and Molecular Medicine, NTNU Norwegian University of Science and Technology, NO-7491 Trondheim, Norway; PROMEC - Proteomics and Modomics Experimental Core Facility at NTNU and the Central Norway Regional Health Authority, NO-7491 Trondheim, Norway; Department of Clinical and Molecular Medicine, NTNU Norwegian University of Science and Technology, NO-7491 Trondheim, Norway; PROMEC - Proteomics and Modomics Experimental Core Facility at NTNU and the Central Norway Regional Health Authority, NO-7491 Trondheim, Norway; Department of Clinical and Molecular Medicine, NTNU Norwegian University of Science and Technology, NO-7491 Trondheim, Norway; Department of Clinical and Molecular Medicine, NTNU Norwegian University of Science and Technology, NO-7491 Trondheim, Norway; Cancer Clinic, St. Olav's Hospital, Trondheim University Hospital, NO-7006 Trondheim, Norway; Department of Clinical and Molecular Medicine, NTNU Norwegian University of Science and Technology, NO-7491 Trondheim, Norway; BioCore - Bioinformatics Core Facility at NTNU and the Central Norway Regional Health Authority, NO-7491 Trondheim, Norway; Institut de Recherches Cliniques de Montréal, 110 Av des Pins Ouest, Montréal, QC H2W 1R7, Canada; Institut de Recherches Cliniques de Montréal, 110 Av des Pins Ouest, Montréal, QC H2W 1R7, Canada; Département of Médicine, Université de Montréal H3C 3J7 Montréal, Québec, Canada; Department of Clinical and Molecular Medicine, NTNU Norwegian University of Science and Technology, NO-7491 Trondheim, Norway; PROMEC - Proteomics and Modomics Experimental Core Facility at NTNU and the Central Norway Regional Health Authority, NO-7491 Trondheim, Norway; Clinic of Laboratory Medicine, St. Olav's Hospital, Trondheim University Hospital, NO-7006 Trondheim, Norway; Department of Clinical and Molecular Medicine, NTNU Norwegian University of Science and Technology, NO-7491 Trondheim, Norway; Clinic of Laboratory Medicine, St. Olav's Hospital, Trondheim University Hospital, NO-7006 Trondheim, Norway

## Abstract

Activation-induced cytidine deaminase (AID) interacts with replication protein A (RPA), the major ssDNA-binding protein, to promote deamination of cytosine to uracil in transcribed immunoglobulin (*Ig*) genes. Uracil-DNA glycosylase (UNG) acts in concert with AID during Ig diversification. In addition, UNG preserves genome integrity by base-excision repair (BER) in the overall genome. How UNG is regulated to support both mutagenic processing and error-free repair remains unknown. UNG is expressed as two isoforms, UNG1 and UNG2, which both contain an RPA-binding helix that facilitates uracil excision from RPA-coated ssDNA. However, the impact of this interaction in antibody diversification and genome maintenance has not been investigated. Here, we generated B-cell clones with targeted mutations in the UNG RPA-binding motif, and analysed class switch recombination (CSR), mutation frequency (*5′ Ig Sμ*), and genomic uracil in clones representing seven *Ung* genotypes. We show that the UNG:RPA interaction plays a crucial role in both CSR and repair of AID-induced uracil at the *Ig* loci. By contrast, the interaction had no significant impact on total genomic uracil levels. Thus, RPA coordinates UNG during CSR and pre-replicative repair of mutagenic uracil in ssDNA but is not essential in post-replicative and canonical BER of uracil in dsDNA.

## Graphical Abstract

**Graphical Abstract:**
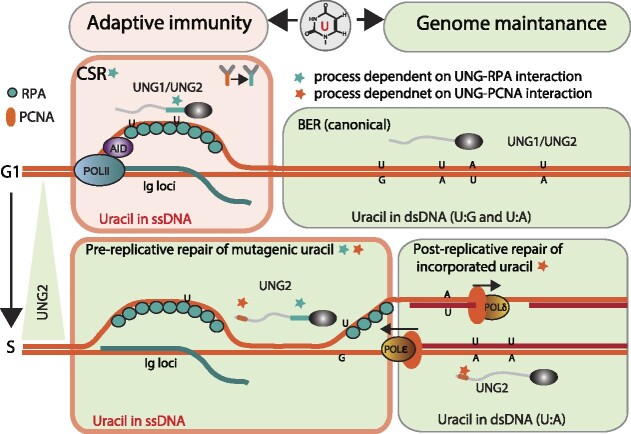

## Introduction

Uracil in DNA is both a general mutagenic lesion and a B-cell specific intermediate in the immunoglobulin genes during antibody diversification (1). Uracil is naturally present in the genome at low levels. In proliferating cells, most uracil sites are likely the result of replicative incorporation of dUMP instead of dTMP (2,3). These uracil sites are not miscoding or directly responsible for inducing mutations. However, uracil in the genome can also arise from deamination of cytosine. Unlike incorporated uracil (U:A), uracil resulting from cytosine deamination is 100% miscoding (U:G), leading to C > T mutations if not repaired prior to replication. In the following we will thus refer to uracil derived from deaminated cytosine as ‘mutagenic uracil’. Cytosine deaminates spontaneously, although at a low rate and preferentially in ssDNA (4). In addition, DNA deamination is catalysed by the AID/APOBEC family of cytidine deaminases, which are important in both adaptive and innate immune responses. In stimulated B cells, activation-induced cytidine deaminase (AID) deaminates cytosine in specific regions of the immunoglobulin (*Ig*) genes to initiate class switch recombination (CSR) and somatic hyper mutation (SHM), which constitute the secondary antibody diversification mechanisms (5). Moreover, several of the APOBEC enzymes inactivate virus by DNA deamination (6). Although genomic uracil is an important intermediate in immunity, it is also a threat to genome stability, and uracil caused by untargeted AID/APOBEC activity is considered a major source of cancer-associated mutations (7).

AID/APOBEC enzymes deaminate cytosine exclusively in ssDNA contexts (8). However, cellular ssDNA is coated with replication protein A (RPA) and AID interacts with RPA to promote deamination of its target sequences in transcribed *Ig* loci (9). The RPA trimer (RPA1, RPA2, RPA3) is also central in replication and genome maintenance, where it protects ssDNA and acts as a binding platform for various protein factors, including the uracil-DNA glycosylase UNG (10–13).

Mammalian cells express several uracil-DNA glycosylases (UNG, SMUG1, TDG and MBD4), but UNG is responsible for most of the uracil excision activity in proliferating cells (14). UNG removes mis-incorporated uracil (U:A) immediately behind the moving replication fork, as well as uracil (U:A and U:G) throughout the genome and induces post-replicative- and canonical BER, respectively. In addition, UNG efficiently excises uracil from ssDNA substrates, at least *in vitro* (14,15).

UNG acts in concert with AID during CSR and SHM. The essential role of UNG in adaptive immunity is demonstrated by the CSR-deficient phenotypes of UNG knockout (KO) mice and human patients with inactivating UNG mutations (16,17). Thus, UNG initiates both error-free base excision repair (BER) of uracil in the genome and mutagenic uracil processing of uracil in *Ig* regions during antibody maturation. How this is regulated to achieve the opposite outcomes is still unknown.

**Figure 1.**
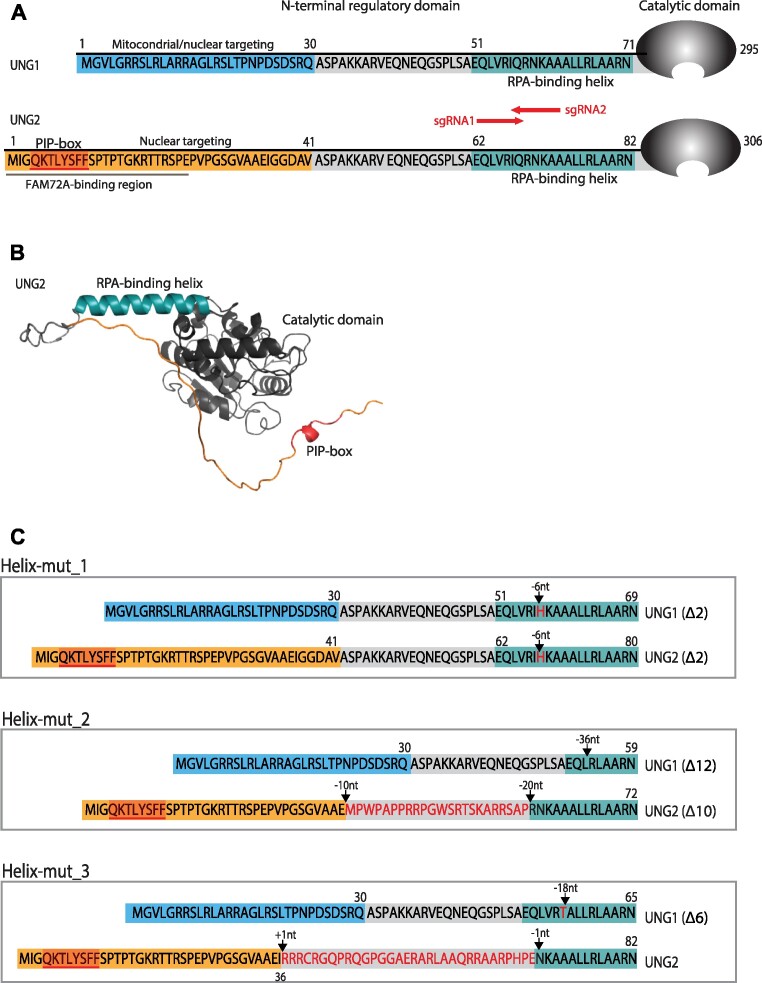
N-terminal domains of mouse UNG isoforms and characterisation of selected CRISPR/Cas clones with targeted mutations in the RPA-binding helix. (**A**) Sequence of the N-terminal domains of UNG1 and UNG2 with the RPA-binding helix adjacent to the catalytic domain. UNG1-specific residues (1–30) mediating mitochondrial and nuclear targeting are marked in blue. UNG2-specific residues (1–41) with the PCNA-interacting peptide (PIP) box motif and FAM72A (Ugene) binding region are in orange. Position and direction of sgRNAs used to mutate the RPA-binding helix in both isoforms are indicated. (**B**) AlphaFold predicted structure of mouse UNG2. The figure was generated in PyMOL2 with coordinates AF-P97931-F1-mod. Colouring of motif residues are as in (A). (**C**) N-terminal domain sequence of UNG1 and UNG2 in the selected clones with in-frame indels in the RPA-binding helix motif. Amino acid sequences are deduced from NG sequencing (Table 1).

UNG is expressed as two major isoforms, UNG1 and UNG2 that differ in their N-terminal sequences (Figure 1A) and regulation. The nuclear UNG2 isoform is downregulated in G1 by FAM72A induced proteolytic degradation and upregulated in S-phase (18–20). UNG2 binds PCNA and associates with the replisome during DNA synthesis (21). By contrast, UNG1 is constitutively expressed during the cell cycle, lacks the PCNA-interacting peptide (PIP) motif, and is targeted to both mitochondria and the nucleus (19). Despite these differences, UNG isotype-specific knockout (KO) B-cell clones, expressing only UNG1 or only UNG2, both display normal genomic uracil levels and Ig class switching activity (19).

In contrast to the UNG2 isoform-specific PCNA- and FAM72A binding sites, both UNG1 and UNG2 contain the helix motif that interacts with the winged-helix (WH) domain of RPA2 (Figure 1A and B). Recently, we demonstrated that this interaction facilitates uracil excision from RPA-coated ssDNA (13). Based on this we suggested that the UNG:RPA interaction may have implications for both repair of mutagenic uracil at the replication fork and mutagenic processing of AID-generated uracil at transcribed *Ig* switch regions to facilitate CSR.

Here we have explored this hypothesis by generating CH12F3 B-cell models with targeted genomic mutations in the UNG RPA-binding helix to investigate the biological function of the interaction. Moreover, we have analysed the individual UNG isoform's ability to repair AID-induced mutagenic uracil. The various cell clones generated were characterised and analysed with respect to Ig class switching (CSR), genomic uracil levels, and repair of AID-induced mutagenic uracil in the *Ig 5′S*μ loci. These experiments reveal that both CSR and repair of mutagenic uracil completely depend upon RPA-mediated UNG recruitment to uracil in ssDNA. By contrast, total genomic uracil was not increased in the mutants lacking RPA-binding, demonstrating that UNG-induced base excision repair (BER) of uracil in the overall genome is independent of RPA. While both UNG isoforms support Ig switching, repair of mutagenic uracil in the *Ig* loci was only executed by the PCNA- and RPA-binding UNG2 isoform. Thus, like switching, repair of mutagenic uracil completely depends on UNG:RPA interaction. Due to the UNG2 dependency, repair of mutagenic uracil in ssDNA likely occurs at the replication fork during S-phase, by an RPA-coordinated mechanism as suggested previously (13).

Based on these results we conclude that RPA guides UNG (not isoform-specific) to uracil in ssDNA in transcribed switch regions to support CSR. Moreover, RPA recruits UNG2 (isoform-specific), which is already in the replisome by its interaction with PCNA, to uracil in ssDNA template strands in replication forks. A likely scenario is then that UNG2 converts uracil to abasic sites that arrest replication and delay repair until dsDNA conformation is restored by a fork reversal or recombination mechanism (13). The unchanged genomic uracil level in clones expressing UNG that lacks RPA-binding demonstrates that normal BER of uracil in dsDNA is independent of RPA. However, we here show that RPA is the key player that orchestrates the functions of UNG in transcribed *Ig* loci during antibody maturation, as well as in repair of mutagenic uracil in ssDNA templates at the replication fork. These results demonstrate the essential role of RPA as well as UNG isoforms to facilitate and regulate the balance between mutagenesis in adaptive immunity and repair to preserve genome stability.

## Materials and methods

### B-cell line and primary splenic B cells from mice

Cells were cultured at 37°C and 5% CO_2_. CH12F3 (22) cell clones were cultured in RPMI-1640 (Sigma) supplemented with 2 mM l-glutamine (Sigma), 10% FCS (heat-inactivated at 56°C for 30 min), 50 μM 2-β-mercaptoethanol (Gibco), and 1 × PenStrep solution (Gibco). U2OS cells were cultured in DMEM–high glucose (Sigma), 2 mM l-glutamine (Sigma), 10% FCS, 1 × PenStrep solution and 1.25 μg/ml Amphotericin B (Sigma). Wildtype, *Ung*^−/−^ (23), *Aicda*^−/−^ (24) mice were in C57BL6/J background (bred for >10 generations). The mice were housed at the IRCM specific pathogens-free animal facility. Mouse work was reviewed and approved by the animal protection committee at the Instituts de Recherches Cliniques de Montreal (protocol 2015–10). Naïve resting B cells were isolated from spleens from three nine to ten-month-old mice using EasySep™ Mouse B cell Isolation kit (STEMCELL) according to the manufacturers’ instructions. The isolated B cells were cultured in RPMI-1640 supplemented with 2 mM l-glutamine, 10% heat-inactivated FCS, 1 mM sodium pyruvate, 50 μM 2-mercaptoethanol, and 1 × PenStrep and stimulated with 10 μg/ml LPS (from *E. coli* strain 0111:B4, Merck) and 40 ng/ml IL-4 (PeproTech).

### Generation of UNG gene edited CH12F3 clones by CRISPR/Cas

Single guide RNAs (sgRNAs) were designed using the CRISPR design tool from the F. Zhang laboratory (http://crispr.mit.edu). Two sgRNAs targeting coding (sgRNA1) and non-coding (sgRNA2) strands were selected and used together to favour deletion mutations in the UNG RPA-binding helix (Figure 1A). 5′-phosphorylated DNA oligonucleotide (Merck) pairs (Supplementary Table S1) were annealed and cloned into the *BbsI* site of the pX458 vector (Addgene, plasmid #48138). One million CH12F3 cells from each of three different founder clones (Table 1) were transfected with a mix of equal amounts of sequence verified pX458-sgRNA1- and pX458-sgRNA2 plasmids (5 μg total) using an Amaxa Nucleofector II device, according to the manufacturer's instructions. GFP-positive cells were sorted by a FACSAria™ II (BD Biosciences) cell sorter 48 h post transfection. Cells were cultured for five days and subcloned by single-cell sorting in 96-well plates. Isotype-specific UNG-KO variants were generated with sgRNA ID, used previously (19). The guide DNA oligonucleotide pairs were annealed and cloned into the pX458 vector. Sequence verified plasmids (5 μg) representing the various guide IDs were used to transfect one million cells. GFP positive cells were sorted and subclones as described above.

**Table 1.** CH12F3 UNG-helix mutated clones with description of founder clones and the editing events generated in this study as revealed by next generation (NG) sequencing

CH12F3	Founder	UNG status founder clone	Helix mutation	NGS reads
UNG mutants	clone #	Indels (nt); target aa	Indels(nt)	(% of total)
Helix-mutant_1	1	WT	−6	78
Helix-mutant_2	2*	U2-KO (−10/−10); 33A/35E	−36/−20	37/30
Helix-mutant_3	3**	U2-KO (+1); 36I	−18/−1	42/37

*Ung2.1-A2 and **Ung2.1-A9 (19); insertion (+); deletion (–); alleles separation (/).

### Sequence verification of targeted genome editing events

Genomic DNA was isolated from the subclones by magnetic nanoparticles in 96-well format using NAxtra™ nucleic acids extraction kit (Lybe Scientific) and the KingFisher™ Flex automated purification system (Thermo Scientific). Barcoded amplicons for next generation (NG) sequencing were generated in two steps. The first PCR reactions were performed with genomic DNA using primers and conditions given in Supplementary Table S1 and S3, which are compatible with our in-house 96-well 6-nt-tag barcoding system. Second PCR reactions (same program) were performed with diluted PCR products as template (1 μl 1:10 diluted) and barcoded primer pars specific for each well in a 96-well plate. All PCR steps were performed with Q5^®^ Hot Start High-Fidelity 2X Master Mix (NEB), according to the manufacturer's instructions. All amplicons (∼200 bp) from each 96-well plate were pooled and purified by Quiaquick PCR purification kit (Quiagen), ∼1 μg DNA from each plate was pooled and sent to Eurofins Genomics for multiplexed amplicon sequencing (150 bp PE). Sequencing data were analysed in-house implementing CRISPResso, a computational pipeline for the analysis of CRISPR/Cas9 genome editing outcomes from deep sequencing data (25). Unique combinations of forward and reverse barcoded primers were used to demultiplex the sequencing data.

### Preparation of cell extracts

Whole cell extracts were prepared by suspending cell pellets in one packed cell volume (PCV) of buffer I (10 mM Tris-HCl pH 8, 200 mM KCl) followed by addition of one PCV of buffer II [10 mM Tris–HCl pH 8, 200 mM KCl, 2 mM EDTA, 40% glycerol, 0.5% NP-40, 2 × cOmplete^®^ protease inhibitor (Roche), 2 mM DTT]. The mixture was rocked at 4°C for 1 h and cell debris was removed by centrifugation at 16000 *g* (10 min). All extracts were snap frozen in liquid nitrogen and stored at 80°C. Protein concentrations were measured using the Bradford assay (Bio-Rad).

### UNG enrichment and detection by UGI pull-down and western blot analysis

The UNG-specific inhibitor UGI (26) was covalently coupled to epoxy beads (Dynabeads™ M-270 Epoxy, Thermo Fisher) as described by the producer. Coupled beads (15 μl) were added to whole cell extract (0.5–1.0 mg total protein) and incubated with shaking at 4°C, overnight. The beads were washed three times with 10 mM Tris-HCl (pH 7.5), and proteins eluted in LDS loading buffer with reducing agent. Proteins were separated by PAGE before western blot analysis of UNG variants using polyclonal rabbit Ab raised against mouse UNG2 (batch 6105, custom-made) and HRP-conjugated swine anti-rabbit IgG (Dako). Bands were visualised in ChemiDoc™ Imager (Bio-Rad).

### Commercial antibodies for Western blot analysis

Primary antibodies: Monoclonal rat anti-AID (Active Motif, 39886); Monoclonal mouse anti-β-Actin (ab8226); Polyclonal rabbit anti-GFP (ab290); Monoclonal rabbit anti-RPA2 [EPR2877Y] (ab76420): Polyclonal rabbit anti-PCNA (ab18197). Secondary antibodies: HRP-conjugated swine anti-rabbit IgG (Dako); HRP-conjugated goat anti-mouse IgG (Dako); HRP-conjugated goat anti-rat IgG (Cell Signaling); IRDye 800CW Goat anti-rabbit (LI-COR); IRDye 680RD goat anti-rabbit (Li-COR); IRDye 680RD goat anti-mouse (Li-COR).

### Uracil excision assays

Uracil excision activity was analysed as described (13). Briefly a 3′-FAM-labelled oligonucleotide (5′-CCACCCCCC**U**CCCCCCCCCCCCCCC-FAM), with a single uracil, was used as substrate. Assays were performed at 22°C with 100 nM DNA substrate (+ 500 nM RPA when indicated) and indicated amounts purified recombinant UNG or 10 μg whole cell extract in assay buffer containing 10 mM Tris–HCl pH 7.5, 50 mM NaCl, 1 mM DTT, 0.1 mM EDTA and 0.5 mg/ml BSA. Reactions were quenched and AP sites cleaved in 10% piperidine at 90°C for 20 min. Samples were dried by vacuum centrifugation and suspended in formamide-containing loading buffer. Product (15 nt) and substrate (25 nt) were separated on 12% PAGE-7M urea gels in 0.5 × TBE buffer. Bands were visualised in a ChemiDoc™ Imager (Bio-Rad) and quantified in Image Lab v6.0.1 (Bio-Rad).

### Site-specific mutagenesis, transfection, and confocal microscopy

Plasmid constructs encoding mUNG1-CFP, mUNG2-CFP, mUNG2-YFP and YFP-RPA2 were described previously (19). The Helix-mut_1 mutation (6 nt deletion) was introduced into the UNG-expressing constructs by the Q5^®^ Site-Directed Mutagenesis Kit (NEB), with forward (acaaggccgcggcgctgctcag) and reverse (ggatgcggacgagctgctcggc) primers, according to the manufacturer's instructions. All mutans were verified by DNA sequencing (Eurofins Genomics Sanger sequencing Service). U2OS cells were transfected with FuGENE^®^ HD or X-tremeGENE™ HP (Roche), 10 mM hydroxyurea (final) was added one day post-transfection to induce visible RPA foci, and cells were examined after 24 h in a Zeiss LSM 510 laser scanning microscope with a Plan-Apochromat 63×/1.4 oil immersion objective. CFP was excited at 458 nm and detected at 470–500 nm, YFP was excited at 514 nm and detected between 530–600 nm.

### RPA and PCNA pull-down

Pull-down experiments were performed with polyclonal GFP antibody (made in house) covalently coupled to Protein A Dynabeads™ (Invitrogen) and whole cell extracts prepared from transfected U2OS cells expressing various tagged UNG variants, as described (19). Briefly, 5 μl GFP Ab-couped beads were mixed with cell extracts (500 μg) in a total volume of 200 μl at 4°C overnight with rotation (1100 rpm). Pull-down of PCNA was performed with nuclease (RNaseA + OmniCleave™) treated extracts. Beads were washed 3 times with 10 mM Tris pH 7.5, and proteins were eluted in LDS buffer and analysed by western blotting.

### Expression and purification of recombinant proteins

Cloning of mouse UNG2 cDNA into the bacterial 6 × His expression vector pET28a was published previously (27). Helix-mut_1 was introduced into the mUNG2-pET28a construct by site-directed mutagenesis as described above. Recombinant mUNG2 proteins (WT and Helix-mut_1) were expressed and purified as described (27). Cloning, expression, and purification of RPA trimer (p11d-tRPA), RPA trimer lacking the RPA2 WH domain (p11d-tRPAΔWH) and the separate RPA2 WH-domain (pTYB12-WH) were described previously (12,13).

### MicroScale thermophoresis (MST)

Recombinant RPA trimer, the WH domain, mUNG2-WT, and mUNG2 Helix-mut_1 were labelled on cysteines and purified using the Monolith NTTM Protein Labelling RED-MALEIMIDE 2nd generation kit (NanoTemper Technologies) according to the manufacturer's protocol. MST was performed on Monolith NT.115 (NanoTemper Technologies) using premium coated capillaries with 40% MST power and 20% or 40% excitation power in MST buffer (50 mM Tris–HCl pH 7.5, 100 mM NaCl, 1mM DTT, 0.05% Tween-20, 0.5 mg/ml BSA). A constant amount of labelled target (100 nM) and a concentration gradient of ligand were used in all experiments. *K*_D_ values were calculated from minimum three runs for each experiment using the MO-Affinity Analysis v2.2.4 software (NanoTemper Technologies).

### Class switch recombination assay

*In vitro* IgM to IgA class switching was measured by flow cytometry. CH12F3 cells (10 000 cells/ml) were seeded and stimulated in flat-bottomed 96-well culture plates in 200 μl growth medium containing 2 μg/ml hamster anti-mouse CD40 (BD Biosciences), 10 ng/ml recombinant murine IL-4 (Peprotech) and 1 ng/ml human recombinant TGF-β1 (PeproTech) (+CIT). Cells were harvested after 72, 96 or 120 h and stained with LIVE/DEAD red stain (Invitrogen), blocked with Fc receptor antibody (2.4G2), fixed and permeabilised in CytoFix/Cytoperm™ and washed in PermWash™ containing saponin. IgA was stained using anti-mouse IgA-PE (eBioscience, 1:200). Cells were washed twice with PermWash™ and suspended in 200 μl PBS before analysis on a FACSCanto™ (BD Biosciences). Viable CH12F3 cells were analysed for IgA expression using FlowJo^®^ version 10 software. Reagents were from BD Biosciences if not stated otherwise.

### Dose response to 5-FdU

Cells (2000 per well) were seeded in 96-well culture plates, cultured for 24 h, and then exposed to various concentrations of 5-fluorodeoxyuridine (5-FdU) (Sigma). Cell viability was measured after 72 h exposure. One volume Resazurin (Sigma) solution (2.5 mM in PBS) was added to five volumes cell culture. The culture plates were incubated at 37°C for 4 h and fluorescence recorded in a FLUOstar® Omega plate reader with excitation wavelength 544 nm and emission wavelength 590 nm.

### Quantification of genomic uracil and 5-fluorouracil by LC/MS/MS

Genomic uracil was quantified as described (19), with some modifications. DNA was isolated from 2 × 10^7^ cells with the Blood & Cell Culture DNA Midi Kit (Qiagen, # 13343) according to the manufactures’ protocol. To minimise risk of uracil excision from DNA during cell lysis and digestion, the UNG inhibitor UGI (30 μg/ml) was added to the lysis buffer. After enzymatic digestion of DNA (30 μg), ^13^C_10_^15^N_2_-dT and ^13^C^15^N_2-_dU internal standards (I.S.) were added prior to dU and dT isolation by LC-UV fractionation. This enables dU and dT quantification in the same fraction, which corrects for possible sample loss during fractionation dU and dT were fractionated using an Agilent Infinity II Analytical-Scale LC-UV Purification System with a mixed mode Primesep 200 column (2.1 mm × 150 mm, 5 μm, SieLC) and mobile phase consisting of water (A) and acetonitrile (B) containing 0.1% formic acid, at 0.4 ml/min and 35°C. The gradient started at 10% B for 1 min, ramping to 60% B in 6 min, followed by 80% B for 2 min and equilibration with 10% B for 9.5 min. Chromatograms were recorded at 260 nm and fractions containing dU, dT, and the corresponding internal standards were collected, freeze-dried, and solved in water for HPLC–MS/MS analysis. Genomic uracil content was calculated as dU/dT and dU/dN ratios. UNG-treated salmon sperm DNA was used as negative control (calculation of detection limit).

Genomic 5-FdU was quantified from 1 μg DNA digested as previously described (28), and dissolved in I.S. solution for LC-MS/MS analysis. Chromatographic separation was performed using an Agilent 1290 Infinity II UHPLC system with an ZORBAX RRHD Eclipse Plus C18 150 × 2.1 mm (1.8 μm) column protected with an ZORBAX RRHD Eclipse Plus C18 5 × 2.1 mm (1.8 μm) guard (Agilent), and a mobile phase gradient with 10 mM ammonium formate and methanol at 0.22 ml/min and 35°C.

For both dU and 5-FdU analyses, mass spectrometric detection of was performed using an Agilent 6495 Triple Quadrupole system monitoring the mass transitions 245.1/155.1 (5-FdU) in negative ionization mode, and 229.1/113.1 (dU), 252.1/136.1 (dA), 228.1/112.1 (dC); 268.1/152.1 (dG), 243.1/127.1 (dT), 264.1/112.1 (2′-deoxy-2′,2′-difluorocytidine I.S. coeluting with 5-FdU), 232.1/116.1 (^13^C^15^N_2-_dU I.S.), 257.1/136.1 (^13^C_5_-dA I.S.), and 255.1/134.1 (^13^C_10_,^15^N_2_-dT I.S.) in positive ionization mode.

### *Ig* 5′Sμ mutation analysis

Mutation analysis was performed as described (29), with modifications. Cells (10000 and 5000 cells/ml) were seeded, stimulated with CD40 ligand, IL4 and TGF-β (CIT), and harvested after 96 and 120 h, respectively, for isolation of genomic DNA using Qiagen DNeasy Blood &Tissue Kit. A 622 bp region (including primers) located 5′ of Sμ in the locus encoding IgM heavy chain was amplified from 100–500 ng genomic DNA with forward (caccaatggatacctcagtggtttttaatggtgg) and reverse (agcggcccggctcattccagttcattacag) primers, using Q5^®^ Hot Start High-Fidelity 2X Master Mix (NEB), according to the protocol, 35 cycles (98°C, 10 s; 69°C, 10 s; 72°C, 30 s). Amplicons were purified by QIAquick PCR Purification Kit (Qiagen), quantified, and cloned into the pENTER™/D-TOPO™ vector (Invitrogen). Plasmids were isolated with Wizard™ Plus Minipreps (Promega) and sequenced from the M13 forward primer (gtaaaacgacggccag) using Eurofins Genomics Sanger sequencing PlateSeq Service. Mutation analysis was performed on a 565 bp sequence (primer sequence not included) by comparing with reference sequence (mouse Ighm, Gene ID:16019). We used SeqKit (30) for statistics and to filter out sequences that were < 600 bases, and MAFFT (31) for the alignments used to identify base substitutions and deletions. Single nucleotide variants present in unstimulated clones, were considered as heterozygote or homozygote alleles, and were not included as mutations in the analysis.

## Results

### Generation and characterisation of CH12F3 B-cell clones with targeted mutations in the UNG RPA-binding helix

To unravel biological roles of the UNG:RPA interaction, we performed CRISPR/Cas gene editing in CH12F3 cells, a mouse B-cell line that induces AID expression and IgM to IgA class switching upon stimulation (22). Our goal was to generate targeted in-frame deletions/mutations in the *Ung* gene that disrupt RPA interaction while preserving catalytic activity, nuclear targeting, and interaction with other factors, such as binding of UNG2 to PCNA.

To increase the probability of introducing small deletions in the critical region, we performed gene editing using a mixture of two sgRNAs that target the RPA-binding helix in UNG1 and UNG2 (Figure 1). We started with three separate CH12F3 clones, wild type (WT) cells and two UNG2 isotype-specific KO clones (19) (Table 1). After subcloning and sequencing we selected three clones for further analysis. Helix-mut_1, generated from WT cells, was homozygote with a small in frame deletion of six nucleotides (Table 1), resulting in substitution of one (Q > H) and deletion of two (ΔRN) critical residues in the RPA-binding helix (12,13) (Table 2, Figure 1C). The two additional clones (Helix-mut_2 and 3), generated from UNG2-KO cell clones, were both heterozygotes and displayed different editing of the two alleles. They were originally selected because they had one allele encoding UNG1 with in-frame deletion in the preferred region, combined with out-of-frame deletions in the second allele (potential knockout alleles). Based on NGS results, 12 and 6 amino acid residues (36 and 18nt) in the RPA-binding helix domains of UNG1 was deleted in Helix-mut_2 and 3, respectively (Tables 1 and 2, Figure 1C). However, their respective out-of-frame Helix deletions in combination with UNG2-specific exon indel frameshift mutations in the founder clones (Table 1), indicated expression of UNG2 Helix-mutated variants with 23 (Helix-mut_2) and 32 (Helix-mut_3) internal out-of-frame residues in their N-terminal domains, respectively (Figure 1C).

**Table 2.** Amino acid composition of the UNG1 and UNG2 RPA-binding helixes in in the mutated clones

CH12F3	UNG1 RPA-binding helix	UNG2 RPA-binding helix
clone	(WT residues 53–70)	(WT residues 64–81)
Wild type	LVRIQRNKAAALLRLAAR	LVRIQRNKAAALLRLAAR
Helix-mutant_1	LVRI*H*—- KAAALLRLAAR	LVRI*H*—–KAAALLRLAAR
Helix-mutant_2	L———————–RLAAR	*RRSAP*RNKAAALLRLAAR
Helix-mutant_3	LVR*T*————ALLRLAAR	*ARPHPE*NKAAALLRLAAR

Missense mutations in italic; deleted regions as dashed lines.

**Figure 2.**
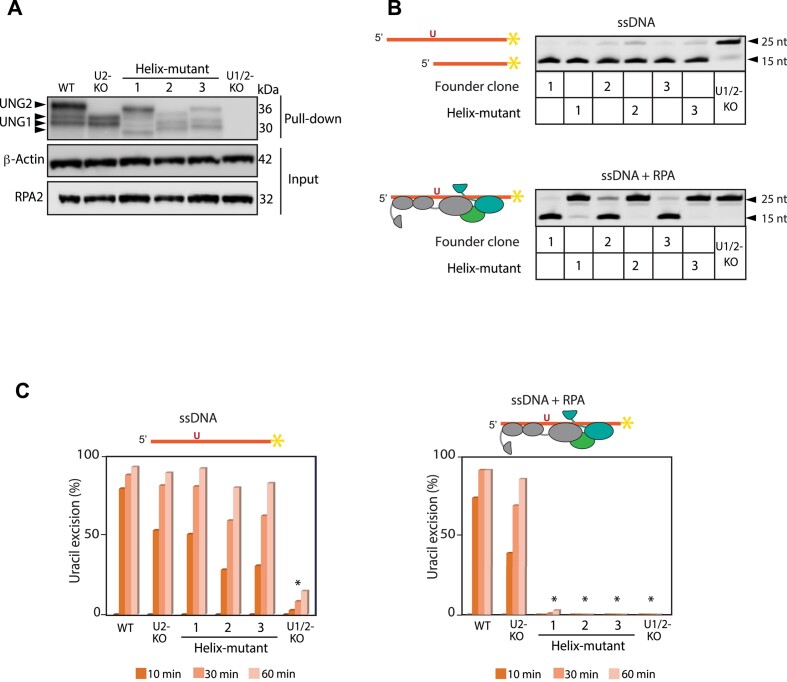
Detection of UNG protein and uracil-excision activity in UNG mutant CH12F3 clones. (**A**) Detection of UNG protein in the mutants, WT (founder clone 1) and UNG2-KO (founder clone 2). UNG1 variants and the UNG2 isoform are indicated by arrows. UNG was affinity-enriched by pull-down using beads coated covalently with the UNG inhibitor UGI before western blot analysis. (**B**) Uracil-excision activity detected in whole cell extracts from mutated clones and their founder clones (Table 1) incubated with ssDNA and ssDNA + RPA substrates for 60 min. Substrate (25 nt) and cleaved product (15 nt) bands are indicated after PAGE separation. Upper panel shows activity with ssDNA as substrate. Lower panel shows uracil-excision activity with RPA-bound ssDNA substrate. Cell extracts from UNG-deficient (U1/2-KO) cells were included as negative controls. (**C**) Quantified uracil-excision activity in whole cell extracts analysed in parallel experiments with the two substrates (ssDNA and ssDNA + RPA) indicated above the panels. Extracts and substrates were incubated in 10, 30 and 60 min before the reactions were stopped. Asterisk (*) indicates significantly different activity compared to WT (*P*< 0.005, Student's T-test, two-tailed, equal variance, paired).

To detect UNG protein expression in the clones, we performed affinity-enrichment of UNG, using UGI-coupled magnetic beads and whole cell extracts, followed by western blot analysis. As predicted by the DNA sequences, we verified UNG1 and UNG2 isoforms in all mutated clones (Figure 2A). For further characterization, we measured uracil excision activity in cell extracts from the mutated clones and their respective founder clones (Table 1), using ssDNA oligonucleotide with a single uracil as substrate and UNG-KO cell extract as negative control. All three Helix-mutated clones displayed uracil excision activity, indicating that the mutations in the N-terminal region of UNG does not inhibit catalytic activity (Figure 2B, upper panel). In our previous study on purified human enzyme variants, we demonstrated that UNG is capable of excising uracil from RPA-coated ssDNA, and that this activity is impaired in UNG mutants deficient in RPA binding (13). To test this in cell extracts from the UNG mutated B-cell clones generated here, we analyzed in the same experiment uracil-excision activity also with RPA-coated ssDNA substrate (Figure 2B, lower panel). In contrast to all founder clones, including UNG2-KOs (founder clones 2 and 3), which efficiently excised uracil from both substrates, cell extracts derived from the three Helix-mutated clones lacked ability to excise uracil from the ssDNA-RPA substrate (Figure 2B). This deficiency, which persisted even after 60 min incubation with the substrates (Figure 2C), strongly indicates that the mutated clones have compromised RPA binding.

For further characterisation, we focused on the homozygote Helix-mut_1 clone that expressed both UNG isoforms with the same deletion mutation (Tables 1 and 2, Figure 1C). We introduced the Helix-mut_1 mutation in CFP- and YFP-mUNG-expressing constructs and investigated cellular localisation and subnuclear co-localisation with RPA by confocal microscopy of transfected U2OS cells. These experiments demonstrate that the Helix-mut_1 deletion does not interfere with either nuclear (UNG2 and UNG1) or mitochondrial (UNG1) import (Supplementary Figure S1A). However, UNG and RPA co-transfected cells show that Helix-mut_1 abolishes accumulation of both UNG isoforms at hydroxyurea-induced RPA foci (Supplementary Figure S1A). The effect of Helix-mut_1 on foci formation was also confirmed in cells co-expressing wild-type (WT) and mutant UNG2, labelled with distinct fluorescent tags (YFP and CFP) (Supplementary Figure S1A, right panel). We also tested Helix-mut_1 in pull-down experiments. In contrast to WT UNG1 and UNG2 isoforms that both efficiently pull down endogenous RPA2, no RPA2 was detected using Helix-mut_1 UNG variants as bait (Supplementary Figure S1B). Importantly, however, PCNA pulldown by UNG2 was not affected by the mutation (Supplementary Figure S1B). These experiments demonstrate that the mutation exerts its effect on the UNG:RPA interaction, without affecting nuclear- and mitochondrial import or binding of UNG2 to PCNA.

For a deeper biochemical characterisation using purified components, we introduced the Helix-mut_1 deletion mutation in a mUNG2 bacterial expression construct (pET28A-mUNG2) (27), and expressed and purified the mutated and WT enzymes. Enzymatic activity assays measuring uracil excision from ssDNA-, ssDNA:RPA-, and ssDNA:RPAδWH- (RPA trimer lacking the UNG-binding WH domain in RPA2) substrates as described (13), demonstrated that the Helix-mut_1 deletion had no impact on enzymatic turnover rate on ssDNA (Supplementary Figure S1C, left panel). However, in agreement with analyses of the cell extract (Figure 2C), it specifically compromised uracil excision from RPA-coated substrates (Supplementary Figure S1C), supporting that the mutation blocks interaction between UNG and RPA.

Finally, we measured direct binding affinity between purified proteins using MicroScale Thermophoresis. In contrast to the mUNG2 WT enzyme, which displayed similar binding affinity (*K_D_* ∼1 μM) to the RPA heterotrimer and the RPA2-WH domain, no binding was detected with mUNG2-Helix-mut_1 (Table 3). By contrast, and in accordance with the pull-down experiments (Supplementary Figure S1B), binding affinity to PCNA was completely preserved in the mutant (Table 3).

**Table 3.** Binding affinity measured by MicroScale thermophoresis (MST)

Labeled target (100 nM)	Ligand	Dissociation constant *K*_D_ (μM)
mUNG2-WT	RPA2-WH	1.26 ± 0.26
mUNG2-WT	RPA-trimer	1.28 ± 0.32
mUNG2-H-mut_1	RPA2-WH	−
mUNG2-H-mut_1	RPA-trimer	−
RPA2-WH	mUNG2-WT	0.86 ± 0.14
RPA2-WH	mUNG2-H-mut_1	−
RPA-trimer	mUNG2-WT	1.26 ± 0.27
RPA-trimer	mUNG2-H-mut_1	−
mUNG2-WT	PCNA	0.16 ± 0.04
mUNG2-H-mut_1	PCNA	0.14 ± 0.04

WH: winged helix domain, *K*_D_ is calculated as mean ± SD from at least three runs.

Thus, based on this thorough characterisation, we conclude that we have constructed an optimal gene-targeted B-cell line model that can be used to reveal the physiological impact of the direct UNG:RPA interaction in Ig class switching (CSR), somatic mutations and repair of genomic uracil.

### Direct UNG:RPA interaction is important for IgM to IgA class switch recombination

Antibody isotype switching is initiated by AID, which deaminates cytosine to uracil in the transcribed *IgH* Sμ locus and a downstream transcriptional activated S region (Sγ, Sα, Sϵ). CH12F3 cells performs IgM to IgA switching upon stimulation with CD40 ligand, IL4 and TGF-β (CIT) (22). In addition, uracil excision by UNG is essential for class switching (16,19,32,33). To investigate whether UNG interacts directly with RPA to promote class switching, we stimulated the three different CH12F3 Helix-mutant clones with CIT for three and four days and measured IgA positive cells by flow cytometry. CIT-stimulated WT and UNG2-KO (isotype-specific) cells were included as positive controls, together with unstimulated WT and UNG-KO (all isoforms) cells as negative controls.

**Figure 3.**
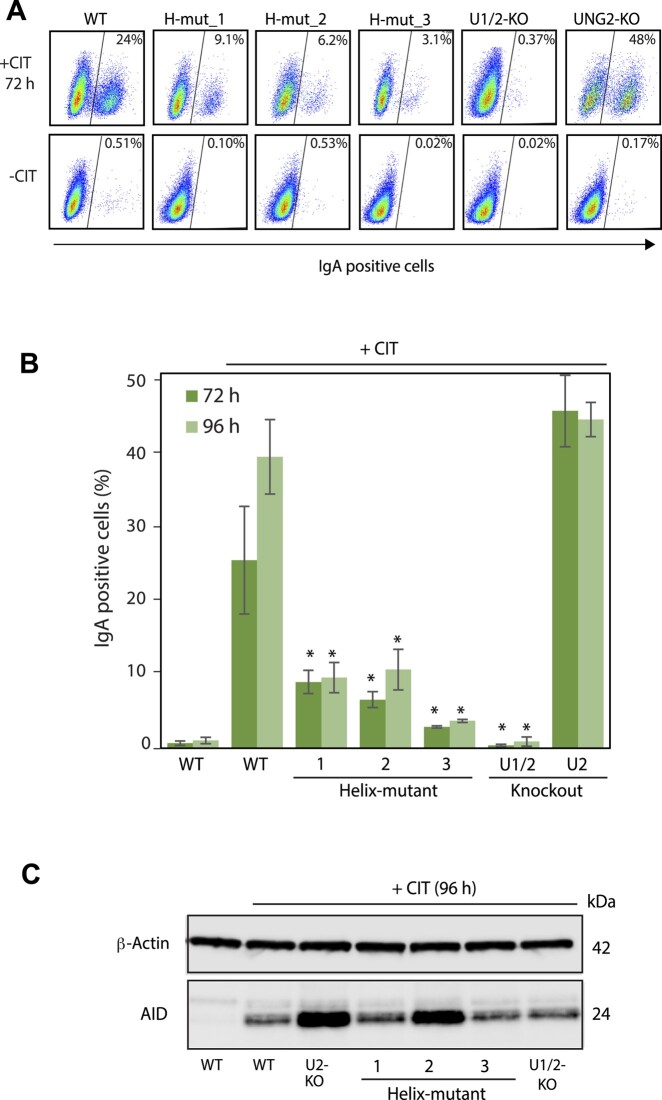
IgM to IgA class switching in UNG mutant CH12F3 clones. (**A**) Representative FACS analysis of CH12F3 clones, showing switching to IgA. Unstimulated (-CIT) cells were included as negative control. Cells were plated and stimulated (+CIT), or not (-CIT), for 72 h before analysis. Fraction IgA positive cells (%) are indicated on each panel. (**B**) Switching activity analysed after 72 and 96 h stimulation. The bars represent the mean of five to eight biological replicates for each condition with standard deviations indicated. WT cells, unstimulated (-CIT) and stimulated (+CIT), UNG2-KO and completely UNG deficient cells (UNG1/2-KO) were included as comparative controls. Asterisk (*) indicates significantly lower CSR activity compared to stimulated WT (*P*< 0.005, Student's T-test, two-tailed, equal variance). (**C**) Western blot showing AID expression in the cell clones. Cells were stimulated (+CIT) for 96 h before analysis. Unstimulated WT cells were included as negative control.

Compared to WT (founder clone 1) and UNG2-KO (founder clone 2) cells, our results show that CSR is strongly compromised in all three Helix-mutant clones, with a reduction in IgA positive cells of ∼80% after four days (96 h) of CIT stimulation (Figure 3A and B). To investigate whether this was due to low AID expression, we subjected cell extracts derived from CIT-stimulated cells to western analysis and found that AID expression in the Helix-mutant clones was not reduced compared to WT (Figure 3C). Based on these results we conclude that appropriate Ig switching depends on the direct interaction between UNG and RPA.

### UNG-induced repair of genomic uracil in dsDNA does not depend on UNG:RPA interaction

Using a quantitative and sensitive LC/MS/MS-based assay, we have previously shown that endogenous AID expression does not significantly increase total genomic uracil in stimulated CH12F3 cells (19), a finding that agrees with a highly regulated and targeted AID activity (34). This also demonstrates that AID-induced cytosine deamination is not a major source to genomic uracil in our cell model. However, using an alternative dot blot-based assay, others have reported AID-dependent increase in genomic uracil in both CH12F3 cells and primary splenic lymphocytes from mice (35). Thus, to ensure that the CH12F3 clones that we have generated here are relevant cell models for primary cells, we also quantified genomic uracil in splenic B cells. Naïve resting B cells from WT, *Ung^−/−^*, and *Ung^−/−^Aicda^−/−^* mice were isolated and stimulated with LPS and IL-4. Cells were harvested each day for four days post stimulation, and genomic uracil measured by the LC-MS/MS method. In accordance with our previous results from CH12F3 cells (19), genomic uracil (dU/dN) increased significantly in *Ung^−/−^* cells compared to WT. However, this increase was independent of AID because genomic uracil levels were not significantly reduced in stimulated B cells from *Ung^−/-^ Aicda^−/−^* compared to *Ung^−/−^* mice (Supplementary Figure S2). This demonstrates that the increase observed after stimulation of naïve resting B-cells is not mediated by AID deamination but likely reflects dUMP incorporation because the cells start to replicate in response to stimulation. Based on this, we conclude that our gene targeted CH12F3 clones are good cell model for replicating cells, including stimulated primary B cells.

**Figure 4.**
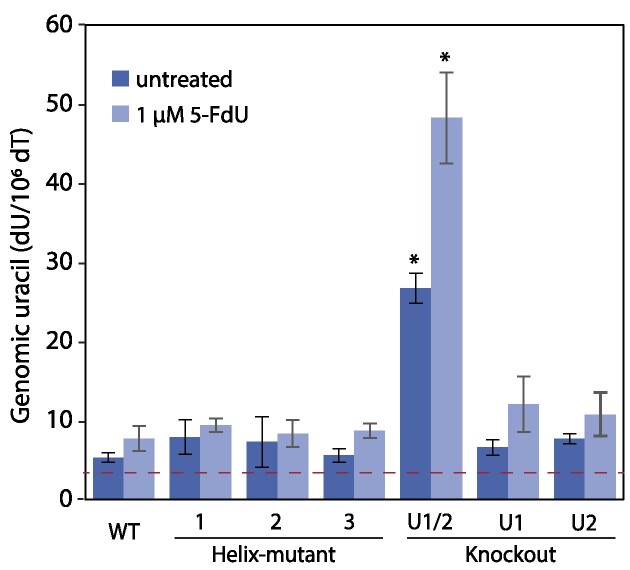
Genomic uracil levels in UNG mutant CH12F3 clones. Genomic uracil (dU/dT) was quantified by LC-MS/MS of hydrolysed DNA (deoxyribonucleosides) in untreated and 5-FdU-treated CH12F3 clones with targeted mutations in UNG. Bars represent mean of three biological replicates with standard deviations indicated. Dashed line indicates detection limit as defined by dU level in UNG-treated control DNA. U1/2-KO is completely UNG deficient, U1-KO is UNG1 deficient but express UNG2, U2-KO is UNG2 deficient but express UNG1. All cell clones were treated (or not treated), and DNA isolated, hydrolysed and analysed in parallel. Asterisk (*) indicates significantly different genomic uracil level compared to similarly treated WT cells (*P*< 0.005, Student's T-test, two-tailed, equal variance).

Because efficient IgM to IgA switching depends on the UNG:RPA interaction (Figure 3), we asked whether disrupting this binding would also reduce the DNA repair (BER) capacity of uracil and thereby increase the overall level of genomic uracil. By measuring dU/dT ratios in hydrolysed genomic DNA, we detected similar levels of uracil in Helix-mutated clones and WT cells (Figure 4). In accordance with our previous study of the UNG isoforms (19), significantly increased genomic uracil was detected only in cells completely devoid in UNG. All cell clones expressing enzymatically active UNG variants displayed WT levels of genomic uracil. This shows that UNG has access to and can excise most of the uracil bases in the genome without interacting with RPA, which is in accordance with that most genomic uracil sites reside in dsDNA context.

To investigate whether this holds true also under conditions of increased replicative incorporation of uracil, cells were treated with the thymidylate synthase (TYMS) inhibitor 5-fluoro-2′-deoxyuridine (5-FdU) to increase the cellular dUTP/dTTP ratio. We performed 5-FdU dose-response experiments to test whether the clones had different sensitivity for the treatment and to ensure that we used conditions (dose and incubation time) not arresting cell proliferation, and thereby replicative incorporation of uracil. All cell clones displayed similar sensitivity for the TYMS inhibitor (Supplementary Figure S3A). Based on these experiments, we decided to treat cells with 1 μM 5-FdU for 24 hours prior to isolation of DNA for genomic uracil analysis. Although genomic uracil modestly increased in all the 5-FdU-treated clones, still only the UNG1/2-KO cells displayed significancy higher levels of genomic uracil compared to WT (Figure 4).

In addition to acting as a TYMS inhibitor, a fraction of 5-FdU is converted to 5-FdUTP in the cells and is incorporated into the genome during replication. Fluorouracil is also a substrate for UNG (28), and an increased level would indicate reduced repair of the lesion. We therefore also measured genomic fluorouracil in WT, H-mut_1 and UNG1/2-KO cells treated with increasing concentrations of 5-FdU for 24 hours. Compared to WT, genomic fluorouracil was significantly increased in UNG1/2-KO cells but not in the RPA-binding deficient UNG Helix mutant (Supplementary Figure S3B, left panel). Moreover, to make sure that we measured repair of incorporated lesions before the next cell cycle S-phase (post-replicative versus pre-replicative), we also did an experiment with cells harvested after only two hours exposure, with similar results. Based on this, we conclude that the UNG:RPA interaction is not essential for the cellular capacity to initiate BER of incorporated lesions (uracil or 5-FU) in dsDNA, which is in agreement with replicative incorporation being the major source of genomic uracil in proliferating cells.

### Repair of mutagenic AID-induced uracil depends on UNG:RPA interaction and the PCNA-binding UNG2 isoform

Whereas replicative misincorporation of dUMP instead of dTMP is not miscoding, uracil from cytosine deamination is 100% miscoding and results in C:G to T:A transitions if not repaired prior to replication. We therefore asked whether RPA is involved in UNG-dependent repair of mutagenic uracil induced by AID deamination.

To study this, we performed *Ig* mutation analysis on a cell clone panel representing UNG WT, Helix-mutants and UNG-KO clones, including UNG isoform-specific KO clones expressing only the UNG1 or UNG2 isoforms. Cells for mutation analysis were harvested after 96 and 120 h stimulation, and genomic DNA representing the various clones and treatment conditions was isolated. DNA isolated from unstimulated cell clones were used as controls. We PCR-amplified the 5′ region of Sμ in the Ig heavy chain locus, cloned the PCR products into a plasmid vector and sequenced ∼50 plasmid clones each from cells stimulated for 96 and 120 h, and ∼10 plasmid clones representing unstimulated cells from each genotype.

**Figure 5.**
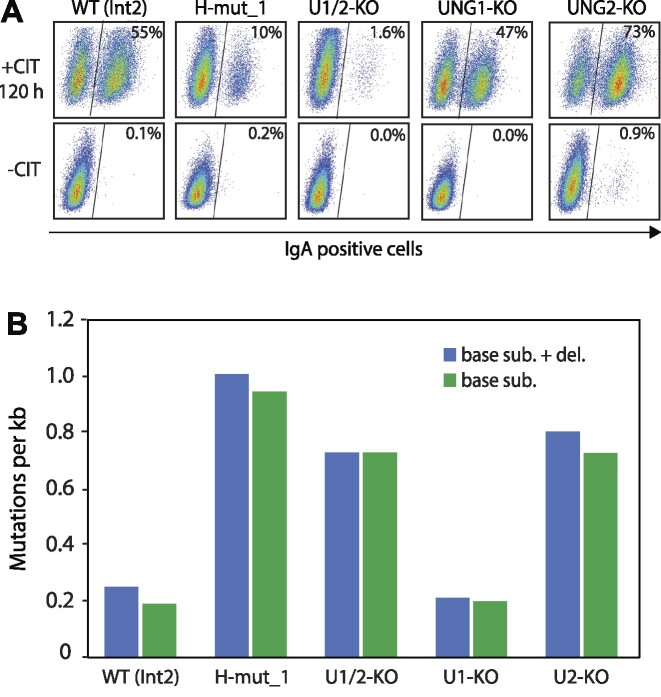
Mutation frequency in *Ig* 5′Sμ region. (**A**) FACS analysis of stimulated (+CIT 120 h) and unstimulated (-CIT) CH12F3 clones, showing IgA switching at harvest in cell cultures used for isolation of genomic DNA and mutation analysis. IgA positive cells (%) are indicated in each panel. (**B**) *Ig* 5′Sμ mutation frequency illustrated as mutations per 1000 sequenced nucleotides (kb). Mutation frequency was calculated from number of mutations identified for each genotype, divided by total number of sequenced nucleotides [# mut./ (# seq. × 565 bases)]. Data are obtained from cells stimulated for 96 h or 120 h, with half of the sequenced plasmid clones representing each condition. Details are given in Table 4 and Supplementary Table S4.

To ensure optimal stimulation of the cells prepared for mutation analysis, IgA-switching (%) was analysed for each culture at harvest. As expected, the various CH12F3 clones displayed switching activity in stimulated (+CIT) and not in unstimulated (-CIT) cultures (Figure 5A, Supplementary Figure S5), with switching rates in accordance with previous analysis (Figure 3B) (19).

The sequencing results demonstrated that a majority of the detected mutations in the *Ig* 5′Sμ regions were C:G to T:A transitions in AID hotspot sequences (Table 4), in accordance with AID-catalysed cytosine deamination as mutation source.

**Table 4.** Summary of mutation analysis of *Ig* 5′Sμ regions in stimulated CH12F3 clones

	Wildtype*		Helix-mut_1		UNG1/2-KO		UNG1-KO**		UNG2-KO***	
	#	(%)	#	(%)	#	(%)	#	(%)	#	(%)
Sequences	177		88		85		197		161	
Mutations	25		50		35		22		73	
Deletions	6	(24)	3	(6)	0		1	(5)	7	(10)
Base subst.	19	(76)	47	(94)	35	(100)	21	(95)	66	(90)
C > T	3	(16)	21	(45)	16	(45)	7	(33)	17	(26)
G > A	15	(79)	19	(40)	15	(43)	8	(38)	43	(65)
GC transv.	1	(5)	5	(11)	2	(6)	3	(14)	6	(9)
AT mutations	0		2	(4)	2	(6)	3	(14)	0	
AID hotspot	18	(95)	39	(83)	25	(71)	13	(62)	49	(74)
Mutations/kb	0.25		1.0		0.73		0.20		0.80	
Base subst./kb	0.19		0.95		0.73		0.19		0.73	

Clones: *WT + Int2; **4B2 + 5B9; ***A2 + A9 (Supplementary Table S4).

**Figure 6.**
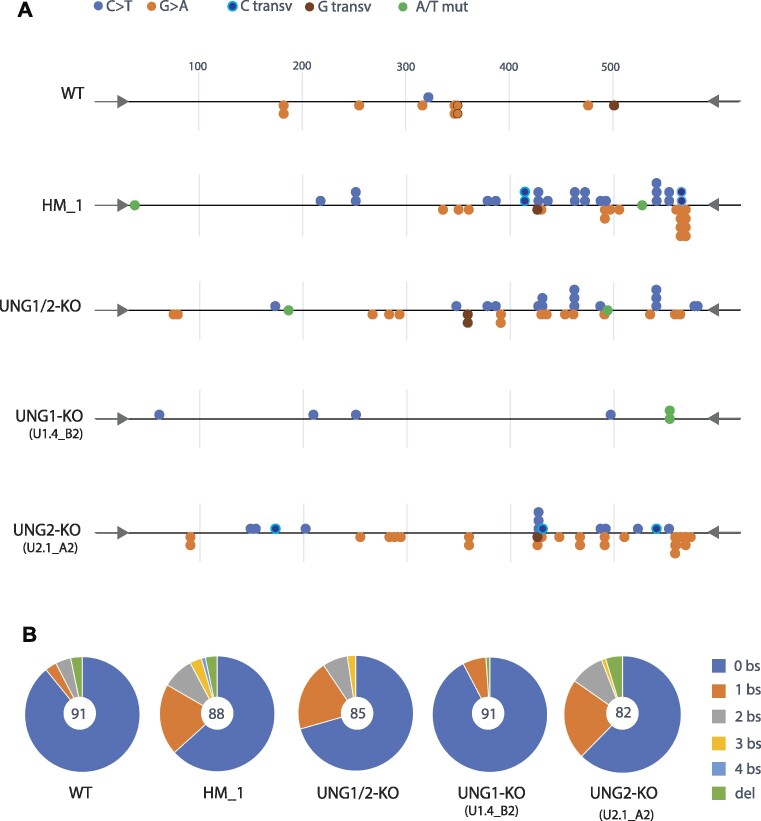
Distribution of mutations in the *Ig* 5′Sμ region. (**A**) Base substitution mutations identified in the various clones, plotted according to localisation and type. Primers are indicated by arrows on each end of the *Ig* 5′Sμ gene fragment. C > T transitions are in accordance with cytosine deamination in non-transcribed strand, while G > A transitions indicate cytosine deamination in the transcribed strand. (**B**) Number of base substitutions (bs) and deletions (del) identified per sequence. Number of sequences per genotype (CH12F3 clone) is indicated in the centre of each diagram (Table 4, Supplementary Table S4).

Interestingly, mutation analyses revealed that the UNG:RPA binding-deficient Helix-mut_1 clone had high AID-induced mutation load, with ∼1 mutation per kilobase (kb). From 88 cloned 565 bp *Ig 5′S*μ sequences, we identified 50 mutations in total, 47 base substitutions and three deletions (Table 4). Mutation frequencies (mutations per kb) were similar in Helix-mut_1- and UNG-deficient cells, which was four-fold higher than in WT (Figure 5B, Table 4 and Supplementary Figure S4). The similarity between Helix-mut_1- and UNG-deficient cells was further verified by comparing the mutation signatures, which were similar both regarding position and type of mutation (Figure 6A), as well as distribution of mutations per sequence (Figure 6B). These results indicate that UNG is dependent on direct interaction with RPA to get access to AID-induced mutagenic uracil and initiate repair.

Surprisingly, the three Helix-mutated clones displayed different mutations frequencies (Supplementary Table S5). We detected higher mutation frequency in Helix-mut_2 than in cells completely devoid of UNG (Table 4) and suspected that this could be caused by high AID expression. To investigate this, we stimulated the Helix-mutated clones and quantified AID protein during a times series of five days (0–120 h) by western analysis (AID/β-Actin normalised to WT control). The results confirmed that mutation frequencies correlated with AID expression in the clones (Supplementary Figure S4). Thus, for the comparative analysis we decided to focus on Helix-mut_1, which was best characterised and expressed AID at a level comparable to WT and UNG-KO cells (Supplementary Figure S1, Figure 3C).

We have previously shown (19), and confirm here that both UNG1 and UNG2 isoforms support class switch recombination and repair incorporated uracil in the genome (Figures 3, 4 and 5A). To investigate whether both isoforms also repair mutagenic uracil in form of deaminated cytosine, we included UNG isotype-specific KO clones in the mutation analysis experiments. The UNG1-KO clones, which express UNG2, displayed a low mutation load (22 mutations in 197 clones) (Table 4, Supplementary Table S4), with mutation frequency like WT cells (Figure 5B). By contrast, in UNG2-KO cells, which still express UNG1, a high number of mutations was identified in the *Ig* 5′Sμ region (73 mutations in 161 clones) (Table 4, Supplementary Table S4), with mutation frequencies like completely UNG-deficient (UNG1/2-KO) and RPA-binding deficient UNG cells (Helix-mut_1) (Figure 5B). Moreover, when comparing the mutation spectra (type, site, mutations per sequence), the mutational footprint in UNG1-KO cells most closely resembled WT cells, while that of UNG2-KO cells resembled complete UNG-deficient (UNG1/2-KO) and RPA-binding deficient cells (Helix-mut-1) (Figure 6A and B).

Taken together, this demonstrates that UNG induced repair of mutagenic uracil, analysed in form of AID deamination sites in *Ig* 5′Sμ, depends on the UNG2 isoform and interaction with RPA. UNG2 is upregulated in S-phase and targeted to the replication machinery by interacting with PCNA, which suggests that repair of AID-induced mutagenic uracil occurs at the replication fork.

### Efficient switching activity mediated by UNG1 is abolished in RPA-binding deficient mutants

**Figure 7.**
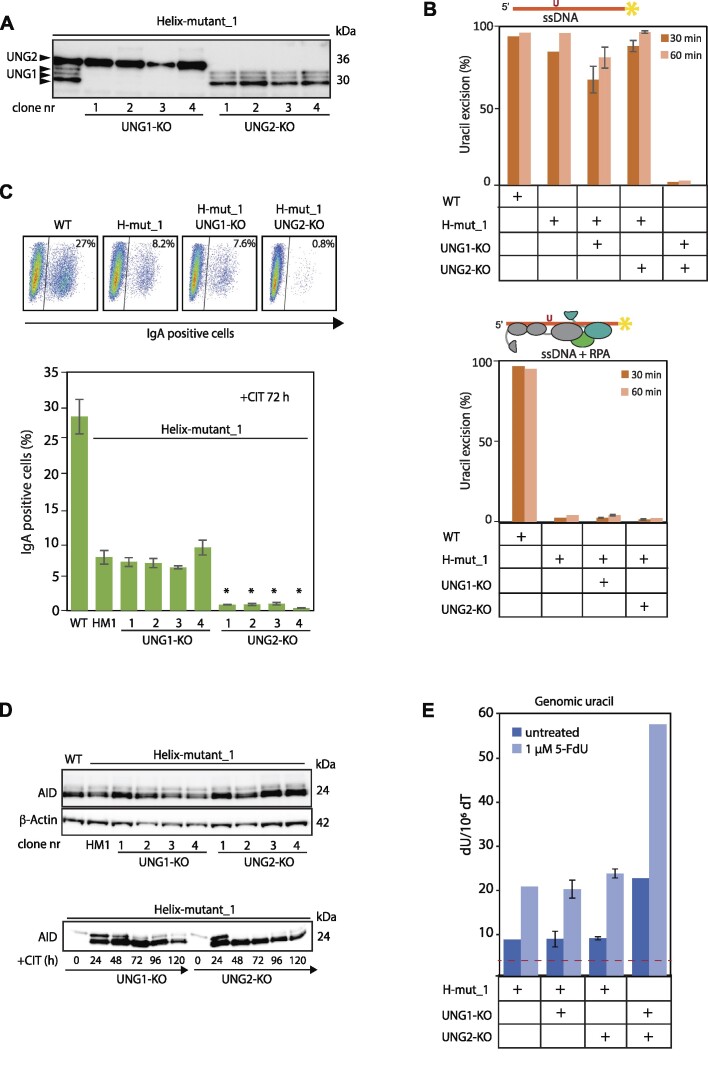
Characterisation of RPA binding-deficient and isotype-specific UNG clones. (**A**) Western blot analysis of Helix-mut_1 UNG isoform knockout clones listed in Table 5. UNG was affinity-enriched by pull-down using beads coated covalently with the UNG inhibitor UGI before western blot analysis. (**B**) Uracil-excision activity in whole cell extracts from double mutated clones compared to founder clone (H-mut_1) and wild type (WT) cells. Extracts were incubated with ssDNA (upper panel) and ssDNA + RPA substrates (lower panel) for 30 min and 60 min. Cell extract from UNG negative cells were included as control. For the H-mut_1 isotype-specific UNG-KO clones, the bars represent mean activity of the four clones representing each genotype in A and Table 5 with standard deviations indicated. (**C**) IgM to IgA class switching in UNG double-mutants. Upper panel: Representative FACS analysis of indicated clones, showing per cent IgA positive cells. Cells were stimulated (+CIT) for 72 h before analysis. Lower panel: Switching activity analysed in four different clones of each H-mut_1 and UNG isoform-specific genotype. The bars represent the mean of three biological replicates with standard deviation as indicated. Asterisk (*) indicates significantly lower CSR activity compared to the H-mut_1 (HM1) founder clone (*P*< 0.005, Student's *t*-test, two-tailed, equal variance). (**D**) AID expression in double mutant clones. Upper: Cell were stimulated with CIT for 24 h and AID expression analysed by western blot. Lower: AID expression level analysed each day for five days in CIT-stimulated UNG1-KO and UNG2-KO cells. (**E**) Genomic uracil in untreated and 5-FdU-treated double mutant clones. H-mut_1 founder clone and UNG negative cell clones were included as controls. Bars represent mean activity of the four clones representing each double mutant in A and Table 5, with standard deviations as indicated.

Although the two UNG isoforms display different cellular localisation and expression profiles during cell cycle (19), both forms repair genomic uracil, interact with RPA and support CSR (Figures 3 and 5A). By contrast, the present work demonstrates that repair of mutagenic uracil at the 5′ Sμ region is mediated exclusively by the PCNA-binding UNG2 isoform (Figures 5, 6). Next, we asked whether the residual switching activities, observed for the RPA-binding deficient UNG mutants (Figure 3A and B), were mediated by UNG1, UNG2 or both. To investigate this, we generated UNG-isoform specific KOs by CRISPR/Cas gene editing in the well-characterised Helix-mut_1 clone. This strategy also yielded a cell model to study the biological function of the UNG:RPA binding helix in absence (UNG2-KO) and presence (UNG1-KO) of the PCNA-binding motif. The new clones were screened by NG sequencing, and based on detection of various frameshift indels, four clones of each isoform-KO variant were selected for further analysis (Table 5). Expression of UNG2 in the Helix-mut_1 UNG1-KO clones and expression of the UNG1 variants in the UNG2-KO clones was demonstrated by western blot analysis, following UNG-enrichment by UGI-coated beads (Figure 7A). Moreover, uracil-excision activity as well as their RPA-binding deficient phenotype were verified by enzymatic assays with naked and RPA-coated ssDNA substrates, respectively (Figure 7B).

**Table 5.** CH12F3 RPA-binding deficient UNG isoform-specific knockout clones as revealed by NG sequencing

CH12F3 clone	Guide ID*	Indels (nt)	First targeted amino acid	Reads (%)
H-mut_1-U1-KO_1	mUng1_4	−29/−2	1M/4L	27/27
H-mut_1-U1-KO_2	mUng1_4	−10	3V	70
H-mut_1-U1-KO_3	mUng1_4	−10/−14	2G/2G	32/32
H-mut_1-U1-KO_4	mUng1_5	–7/–14	12A/12A	36/28
H-mut_1-U2-KO_1	mUng2_1	+1	36I	67
H-mut_1-U2-KO_2	mUng2_1	−13/−10	31G/35E	40/30
H-mut_1-U2-KO_3	mUng2_1	−2	35E	72
H-mut_1-U2-KO_4	mUng2_1	+1/−1	37G/36I	39/34

*(19); insertion (+); deletion (–); alleles separated by (/).

We then analysed IgM to IgA switching in this new panel of UNG isoform-specific RPA-binding deficient mutants. All four UNG1-KO clones displayed switching activity levels like the founder clone (Helix-mut_1). Conversely, in the UNG2-KO clones (expressing RPA-binding deficient UNG1 and normal levels of AID), switching was essentially abrogated (Figure 7C and D). This demonstrates that the residual switching activity observed in the Helix-mutated clones (Figures 3 and 5A), is mediated by the PCNA-binding proficient UNG2 isoform (Supplementary Figure S1B). Importantly, comparing the UNG1 expressing UNG2-KO clone (Figure 3B), which is 100% switching proficient, and the corresponding Helix-mut_1 UNG2-KO clones (Figure 7C), which is 100% switching deficient, underscores the impact of direct interaction between RPA and UNG to support class switching.

We have already shown that cells expressing RPA-binding deficient UNG (both isoforms) are unable to mediate repair of mutagenic uracil induced by AID at the *Ig* loci (Figures 5 and 6). We also verified this by *Ig* mutation analysis in the Helix-mut_1 and UNG isotype-specific double KO clones. Although based on fewer sequenced clones, the results were as expected. Both genotypes (Helix-mut_1-UNG1-KO and Helix-mut_1-UNG2-KO) displayed AID mutation footprints (CG-TA transitions in AID hotspots) with mutation frequencies at levels comparable to completely UNG-deficient cells, the RPA-binding deficient UNG founder clone (Helix-mut_1) and UNG2-deficient clones (Tables 6, 4, Supplementary Table S4).

**Table 6.** Mutation analysis of *Ig* 5′Sμ regions in stimulated RPA-binding deficient isoform-specific UNG CH12F3 clones

	H-mut_1 UNG1-KO		H-mut_1 UNG2-KO	
	#	(%)	#	(%)
Sequences	39		34	
Mutations	17		18	
Deletions	1		0	
Base subst.	16		18	
C > T	7	(44)	11	(61)
G > A	9	(56)	6	(33)
GC transv.	0		0	
AT mutations	0		1	
AID hotspot	13	(81)	11	(61)
Mutations/kb	0.77		0.94	
Base sub./kb	0.73		0.94	

Finally, we quantified genomic uracil levels in the double-KO clones. We isolated DNA from both untreated and 5-FdU-treated cells and included the founder clone (Helix-mut_1) and UNG-KO cells as controls. In addition, we exposed the cell clones with increasing concentration of 5-FdU and measured the level of incorporated fluorouracil in genomic DNA. In contrast to completely UNG-deficient cells, which display elevated genomic uracil and fluorouracil levels, the RPA binding-deficient and UNG isoform-specific double KO clones, expressing either the UNG1 or UNG2 isoform, retained low levels of genomic uracil (Figure 7E) and fluorouracil (Supplementary Figure S6). This demonstrates that repair of incorporated uracil (and fluorouracil) in the genome is neither dependent on UNG isotype nor interaction with RPA.

In summary, by analysing the UNG1 and UNG2 isoforms separately, we confirm that both UNG isoforms can separately support switching by interacting directly with RPA. Cells expressing RPA binding deficient UNG1 are 100% switching deficient. The UNG2 expressing (Helix-mut_1 UNG1-KO) clones, as well as the UNG1 + UNG2 expressing (Helix-mut_1) founder clone, display a low but still detectable residual switching activity, which may be mediated by PCNA-dependent recruitment of UNG2 to dsDNA-ssDNA junctions at switch regions. Differing from the process of switching, which relies on RPA interaction, and the repair of AID-induced mutagenic uracil, requiring both RPA interaction and the involvement of the PCNA-interacting UNG2 isoform, the repair of the majority of uracil sites within the genome, primarily in the form of incorporation into double-stranded DNA (dsDNA), proceeds with remarkable efficiency. Importantly, this repair process occurs independently of UNG isoform variation and interactions with RPA.

## Discussion

**Figure 8.**
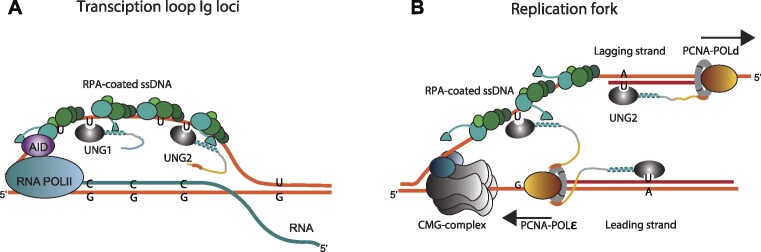
Model showing RPA-dependent targeting of UNG to deamination sites in transcribed *Ig* switch regions and in template strand at the replication fork. (**A**) RPA recruits UNG (both isoforms) to AID-generated uracil in ssDNA in transcribed *Ig* switch regions during G1. Deamination is indicated for the non-transcribed strand only. However, deamination occurs on both strands and processing results in the ds breaks needed to complete class switch recombination. (**B**) In S phase UNG2 bound to PCNA is part of the replisome where it removes incorporated uracil (U:A) in the nascent strand immediately behind the moving fork. The long flexible N-terminal region of UNG2, with PCNA and RPA-binding motifs positioned on each end (Figure 1A, B), can probably bind both factors at the same time. Thus, UNG2 bound to PCNA can be recruited by RPA to excise uracil in the template strand. This will turn the 100% mutagenic uracil (deaminated cytosine) to a blocking AP site that arrests replication and promotes fork reversal, followed by DNA repair and fork restart.

UNG initiates both mutagenic uracil processing at *Ig* loci during antibody maturation in B cells and error-free repair of uracil in the genome in all cell types. The enzyme is expressed as two major isoforms, UNG1 and UNG2 with unique N-terminal extensions that guide subcellular localisation and protein interactions. UNG1 is localised to both nucleus and mitochondria, while UNG2 is only in nucleus where it associates with the replication machinery by binding to PCNA. By contrast, both isoforms interact with the ssDNA-binding protein RPA, which is involved in many biological processes, including replication, recombination, DNA repair and transcription (36,37). To date, cell models to study the physiological function of the UNG:RPA interaction have not been available. Thus, to investigate the role of this interaction in adaptive immunity and DNA repair, we here generated B-cell clones with targeted mutations in the UNG RPA-binding helix. We further explored the functions of the UNG1 and UNG2 isoforms, including the impact of the RPA binding helix in isoform-specific KO clones. By analysing CSR efficiency (IgM to IgA), local repair of AID-induced uracil at *Ig 5′S*μ loci, and global uracil repair (genomic uracil level) in a panel of cell clones representing seven different UNG genotypes (Table 7), we demonstrate that RPA recruits UNG to uracil in ssDNA to initiate both CSR and repair of mutagenic uracil. Thus, RPA is the coordinator of UNG during both antibody class switching and pre-replicative repair of mutagenic uracil, two processes likely initiated by excision of uracil in ssDNA but separated by timing (G1 and S-phase) and biological context (transcription and replication) (Figure 8).

**Table 7.** Summary of biological activity in UNG gene edited CH12F3 clones

CH12F3 clone	WT	H-mut_1	U1/2-KO	U1-KO	U2-KO	H-mut_1	H-mut_1
						U1-KO	U2-KO
Isoforms	UNG1	UNG1	−	−	UNG1	−	UNG1
expressed	UNG2	UNG2	−	UNG2	−	UNG2	−
RPA interaction	**+**	**−**	**−**	**+**	**+**	**−**	**−**
PCNA interaction	**+**	**+**	**−**	**+**	**−**	**+**	**−**
CSR IgM to IgA	**+**	low	**−**	**+**	**+**	low	**−**
Repair *Ig* 5′Sμ	**+**	**−**	**−**	**+**	**−**	**−**	**−**
BER genome	**+**	**+**	**−**	**+**	**+**	**+**	**+**

Interestingly, AID also interacts with RPA to get access to ssDNA at transcribed Ig loci, an interaction that is regulated by phosphorylation on a serine residue in AID (Ser 38) (38,39). Thus, RPA plays a critical role as a coordinating hub during Ig diversification (Figure 8A).

In contrast to CSR, genomic uracil level measurements (Figure 4, Table 7) indicate that the UNG:RPA interaction has no impact on the global uracil repair capacity of the cells. Thus, repair of most uracil sites in the genome (mainly post-replicative and canonical BER of misincorporated uracil in dsDNA), is independent on interaction with RPA. Importantly, we also identified UNG isoform-specific functions. In contrast to CSR and total genomic uracil repair, which are similar in UNG1 and UNG2 single-isoform expressing cells (19), only UNG2 expressing cells repair AID-induced mutagenic uracil in the *Ig* loci (Table 7). This indicates that repair of these mutagenic uracil sites depends on interaction with both PCNA and RPA and that the process occurs during S-phase when UNG2 expression is upregulated. Moreover, the results support a replication fork-associated repair process of mutagenic uracil, as suggested (Figure 8) (13).

The secondary Ig diversification processes are strictly regulated during the cell cycle (40). The importance of low UNG2 expression in G1 to reduce error-free repair of AID-induced uracil and promote antibody diversification was recently shown by two research teams (20,41). They observed that knocking out the UNG2 isoform-specific binding factor FAM72A, originally named Ugene (42), resulted in reduced CSR and SHM due to upregulation of UNG2. Moreover, they showed that FAM72A downregulates UNG2, but not UNG1, by inducing proteolytic degradation in G1 phase of the cell cycle. The cell cycle also plays an important role in regulating AID activity (40,43,44). AID accumulates in the nucleus and deaminates its *Ig* target sequences only in the G1-phase of the cell cycle. By contrast, UNG2 is downregulated in G1 and upregulated in S-phase. Thus, an opposite regulation of AID and UNG2 activity during the cell cycle is likely important to support both mutagenic Ig diversification (CSR and SHM) in G1 and repair of remaining mutagenic uracil sites entering the replication fork in S phase.

In contrast to UNG2 deficiency, which has no impact on switching efficiency (due to the presence of UNG1), overexpression of UNG2 reduces switching by ∼50% in FAM72A KO CH12F3 cells (20,41). This may be explained by the way DNA breaks in switch regions are formed to induce recombination. These dsDNA breaks are generated by UNG in concert with AP-endonuclease (APE1) and mismatch repair (MMR) proteins (MSH2/6, MLH1/PMS2, EXO1). MMR proteins depend on the recognition of U:G mismatches to generate ds beaks. Overexpression of the highly efficient UNG2 enzyme will process almost all AID-generated uracil sites, and thereby reduce the amount of remaining U:G substrates available for MMR, which again compromise formation of dsDNA breaks and CSR, as observed (20,41). This also conforms to the ∼50% reduced switching activity in stimulated B cells from MMR deficient mice (45).

We have previously suggested that the action of UNG during cell cycle, at specific loci or during specific biological processes is regulated by its N-terminal domain and interaction with the coordinating factors RPA and PCNA (13,18,19). PCNA encircles duplex DNA where it guides UNG2 to remove incorporated uracil in the nascent strand directly behind the moving fork by a post-replicative BER mechanism (21). Here we demonstrate that PCNA is involved together with RPA also in pre-replicative repair of mutagenic uracil in the single-stranded template strand (Figure 8B), likely by a mechanism involving AP-site induced fork arrest and reversal, followed by repair and replication restart, as suggested (13). Based on our results we conclude that, in addition to post-replicative repair of incorporated uracil in the nascent strand, PCNA positions UNG2 near the RPA-coated template strand to facilitate removal of mutagenic uracil in ssDNA.

Coordinating UNG function through interaction with RPA and PCNA is regulated at several levels, including expression of the UNG isoforms (amount and UNG1/UNG2 ratio), nuclear and mitochondrial targeting of UNG1, as well as cell-cycle regulated expression, phosphorylation, and protein turnover of UNG2 (18–20,41). Interestingly, phosphorylation at Tyr8 in the UNG2 PCNA-binding site, the most frequent PTM reported in UNG (www.phosphosite.org), directly impedes binding to PCNA (46). Ubiquitination on a central Lys residue (hUNG2 K78, mUNG2 K71) is the only identified PTM in the RPA binding region of UNG (www.phosphosite.org), and recently we showed that this modification stimulated uracil-excision from RPA-coated ssDNA (13). Thus, this demonstrates that the N-terminal region is essential in regulating UNG activity and targeting to fulfil its various roles in antibody maturation and DNA repair.

Interestingly, the isoform-specific functions of UNG may potentially allow B cells to regulate the *Ig* mutation load by suppressing repair of AID-induced mutagenic uracil in G1, without compromising class switching. We have shown previously (19), and confirm here that CSR is not dependent on the UNG2 isoform (Figure 3, Table 7). When UNG2 is downregulated in G1 by FAM72A-induced degradation (20,41), the constitutively expressed UNG1 isoform is still present and can support CSR. However, in contrast to UNG2, UNG1 does not repair mutagenic uracil at *Ig* loci. Thus, without influencing switching efficiency or genomic uracil levels (Figure 4), the mutation load at *Ig* loci can be adjusted to an optimal level by regulating UNG2 level, and thereby repair efficiency during the S-phase. This knowledge may potentially allow development of highly specific UNG inhibitors. It is tempting to speculate that inhibitors that block the UNG:RPA- or UNG:PCNA interaction, or alternatively one of the isoforms, can be used to manipulate the balance between the various functions of UNG. With such strategy it may be possible to increase mutation load (inhibit pre-replicative repair), while CSR activity and global uracil repair by post-replicative and canonical BER are preserved.

In our recently published model on RPA-facilitated repair of mutagenic uracil at the replication fork, we suggested the involvement of the newly discovered HMCES protein (13). HMCES (5-hydroxymethylcytosine binding, embryonic stem-cell-specific) is a suicide enzyme that covalently crosslinks to AP sites in ssDNA (47–49). Interestingly, like UNG, HMCES has a binding site for PCNA (47) located in a predicted flexible terminal region (AlphaFold AF-Q96FZ2-F1) and physically interacts with RPA (www.thebiogrid.org). Moreover, it is targeted to replication forks to maintain genome integrity and to *Ig* loci to support antibody diversification (50–52). Taken together this strongly indicates that UNG and HMCES are guided by RPA to act in concert on ssDNA, and that this occurs both at the replication fork to induce repair of mutagenic uracil and at *Ig* loci to support antibody diversification.

To conclude, the present work explains how the ‘double edge sword’-functions of UNG are fine-tuned to funnel genomic uracil into appropriate mutagenic- or repair pathways. By using functional assays and gene-targeted cell models, we demonstrate that the interaction between UNG and RPA is essential in both antibody class switching and repair of mutagenic uracil in ssDNA. This knowledge completes the picture on how UNG is targeted to various genomic contexts to fulfil its different roles in adaptive immunity and genomic integrity.

## Supplementary Material

gkad1115_Supplemental_File

## Data Availability

The data underlying this article are available in the article and in its online supplementary material.
