# Supplementary material for: RPA guides UNG to uracil in ssDNA to facilitate antibody class switching and repair of mutagenic uracil at the replication fork
Source: Nucleic Acids Res. 2023 Nov 24;52(2):784–800. doi: 10.1093/nar/gkad1115 (PMC10810282; doi:10.1093/nar/gkad1115)
Supplement: gkad1115_Supplemental_File [file gkad1115_supplemental_file.pdf]

SUPPLEMENTARY DATA

RPA guides UNG to uracil in ssDNA to facilitate antibody class switching and repair of mutagenic uracil at the replication fork

Table S1. CRISPR/Cas single-guide DNA oligos

Guide ID	Target gene region	DNA oligo sequence	Forw. (+)/Rev. (-)
sgRNA1	UNG RPA-binding helix	caccgAGCTCCTCCGCATCCAG	+
	mouse *Ung* (exon 1B)	aaacCTGGATGCGGAGGAGCTc	-
sgRNA2	UNG RPA-binding helix	caccgCCGCGGCCTTGTTCCCTC	+
	mouse *Ung* (exon 1B)	aaacGAGGGAACAAGGCCGCGGc	-

Table S2. PCR primers for screening of CRISPR gene editing events in the mouse *Ung* gene region encoding the RPA-binding helix

Primer	Target gene region	5' extensions	Target sequence
mUNG_A+	*Ung* (exon 1B)	cacgacgacatggttccagat	CCCGACTCTGACTCCCGGC
mUNG_B+		cacgacgacatggttccagta	
mUNG_1-	*Ung* (exon 1B)	caccagcaggacgactagcat	CCTTGACGAAGTACGGCTTCCC
mUNG_2-		caccagcaggacgactagcta	

Amplification : (95 °C 30 s, 72 °C 1 min) 34 cycles

Table S3. PCR primers for screening of CRISPR gene editing events in *Ung* isoform-specific exons

Primer	Target gene region	5' extensions	Target sequence
mUNG1_A+	*Ung1*-sp. (exon 1B)	cacgacgacatggttccagat	TTGCCGCGAAAAGCCTGCG
mUNG1_B+		cacgacgacatggttccagta	
mUNG1_1-	*Ung* (exon 1B)	caccagcaggacgactagcat	TTGCTCGTTCTGCTCCACCCG
mUNG1_2-		caccagcaggacgactagcta	
mUNG2_A+	*Ung2*-sp. (exon 1A)	cacgacgacatggttccagat	GCATGATCGGCCAGAAGACCC
mUNG2_B+		cacgacgacatggttccagta	
mUNG2_1-	*Ung* (intron 1B)	caccagcaggacgactagcat	GCAAGCGGCGTGCGTGGC
mUNG2_2-		caccagcaggacgactagcta	

Amplification: (95 °C 30 s, 70 °C 30 s) 34 cycles

Table S4. Mutation analysis of Ig 5'Sµ region in different WT and UNG isoform-specific knockout clones

**Genotype**	**UNG wildtype**		**UNG1-knockout**		**UNG2-knockout**	
Clone	WT	Int2	U1.4_B2	U1.5_B9	U2.1_A2	U2.1_A9
# Sequences	91	86	91	106	82	79
# Mutations	14	11	7	15	47	26
# Deletions	3	3	1	0	4	3
# Base subst.	11	8	6	15	43	23
# C>T	1	2	4	3	10	7
# G>A	9	6	0	8	28	15
# GC transv.	1	0	0	3	5	1
# AT mutations	0	0	2	1	0	0
# AID hotspot	10	8	3	10	33	16
Mutations/kb	0.27	0.23	0.14	0.25	1.0	0.58
Base subst./kb	0.21	0.16	0.12	0.25	0.93	0.52

Table S5. Summary of mutation analysis of Ig 5'Sµ regions in stimulated CH12F3 clones with mutations in the UNG RPA-binding helix

	Helix-mut_1		Helix-mut_2*		Helix-mut_3**	
	#	(%)	#	(%)	#	(%)
Sequences	88		93		95	
Mutations	50		126		16	
Deletions	3	(6)	3	(2)	3	(19)
Base subst.	47	(94)	123	(98)	13	(81)
C>T	21	(45)	40	(33)	5	(38)
G>A	19	(40)	80	(65)	7	(54)
GC transv.	5	(11)	3	(2)	0	
AT mutations	2	(4)	0		1	(8)
AID hotspot	39	(83)	88	(72)	9	(69)
Mutations/kb	1.0		2.40		0.30	
Base subst./kb	0.95		2.34		0.24	

*AID high expression; **AID low expression (Supplementary Figure S4)

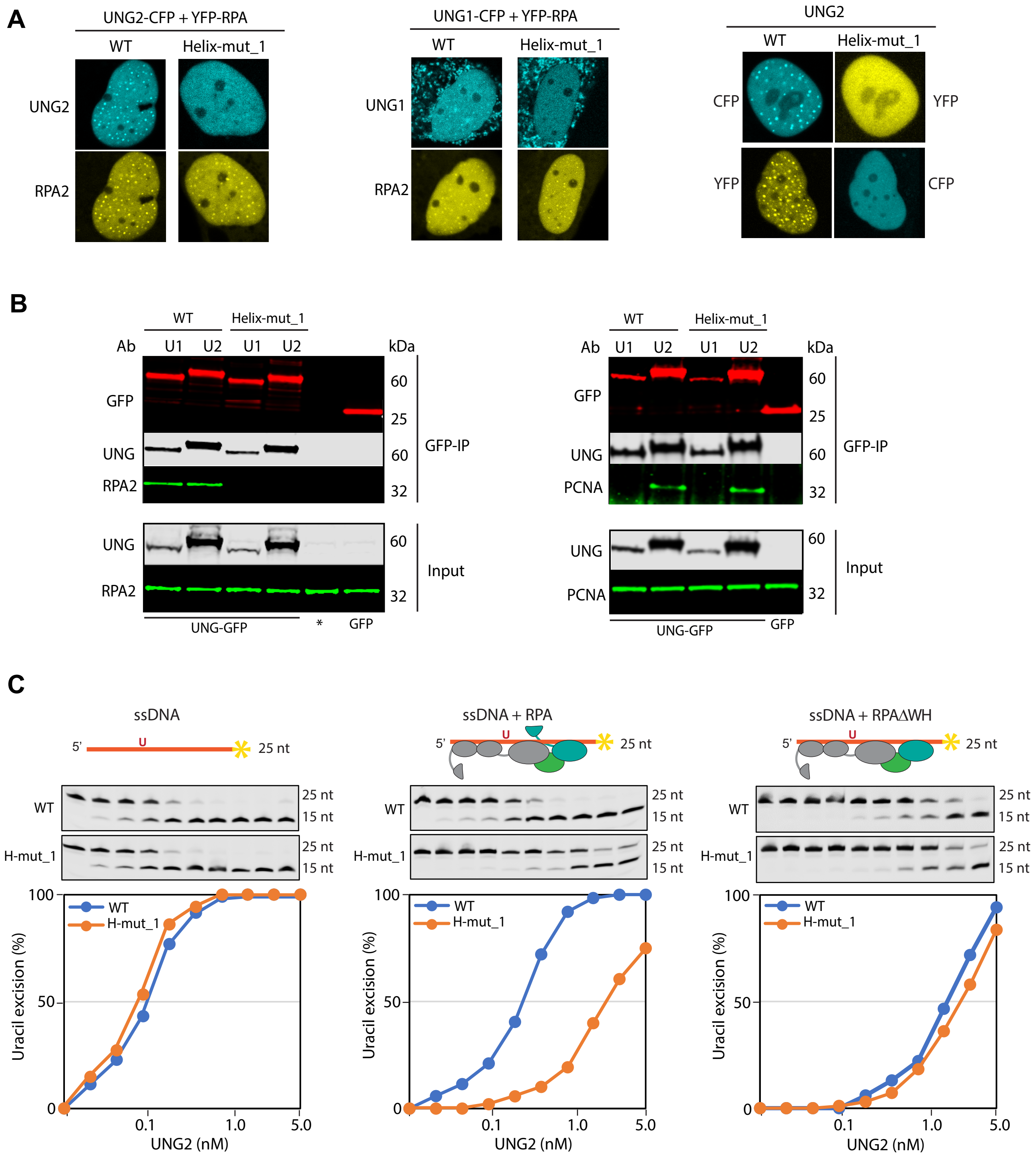

Figure S1. Characterization of Helix-mutation_1

A. Helix-mutation _1 disrupts colocalization of UNG and RPA2. Representative confocal microscopy image of live U2OS cells treated with 10 mM hydroxyurea for 24 hrs to induce visible foci. Cells were co-transfected with, Left panel: UNG2 (WT/mutant) + RPA2, Middle panel: UNG1 (WT/mutant) + RPA, and Right panel: UNG2-WT (CFP/YFP) + UNG2-mutant (YFP/CFP).

B. Helix-mutation _1 disrupts pull down of RPA by UNG1 and UNG2, but not pull down of PCNA by UNG2. U2OS cells were transfected with wildtype UNG1-GFP, wildtype UNG2-GFP, mutated UNG1- GFP and mutated UNG2-GFP as indicated. GFP immunoprecipitation (IP) was performed in whole cell extracts as described in the method section. Pull down of RPA and PCNA were detected by western blot analysis.

C. Helix-mutation_1 prevents access of UNG to uracil in RPA-coated ssDNA. Comparative uracil excision activity analysis of purified recombinant mouse UNG2 wildtype (WT) and mutant (Helix-mutant_1). Increasing amount (0-5.0 nM) of enzyme were incubated with one of the three different substrates, as indicated on top of each panel. RPAΔWH lack the RPA2-WH domain does not bind UNG. The curves in the three panels represent one representative experiment, where all samples were run in parallel using the same enzyme dilutions with the tree substrates. Assays were performed as described in the method section.

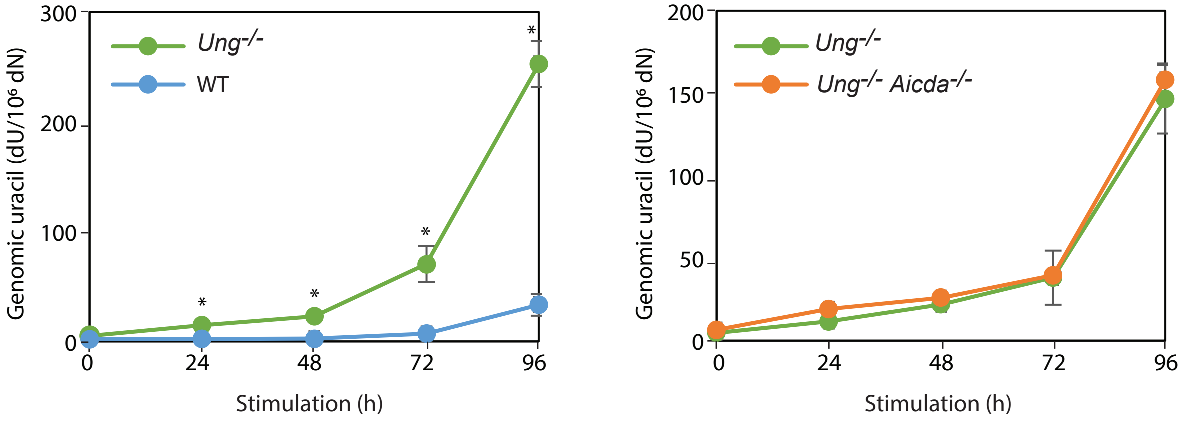

Figure S2. Genomic uracil in stimulated primary splenic B cells from wildtype (WT), *Ung*$^{-/-}$, and *Ung*$^{-/-}$ *Aicda*$^{-/-}$ mice

Naïve splenic B cells were isolated from 9-10-month-old mice of each genotype. Resting B cells were stimulated with LPS and IL-4 for the time intervals indicated. DNA was isolated and genomic uracil quantified as described in the material and method section. Curve points represent mean values from three mice of each genotype with standard deviations indicated. All samples within each panel are prepared and quantified in parallel. Asterisk (*) indicates significantly different genomic uracil level between the genotypes at each time point (P<0.005, Student's T-test, two tailed, equla variance).

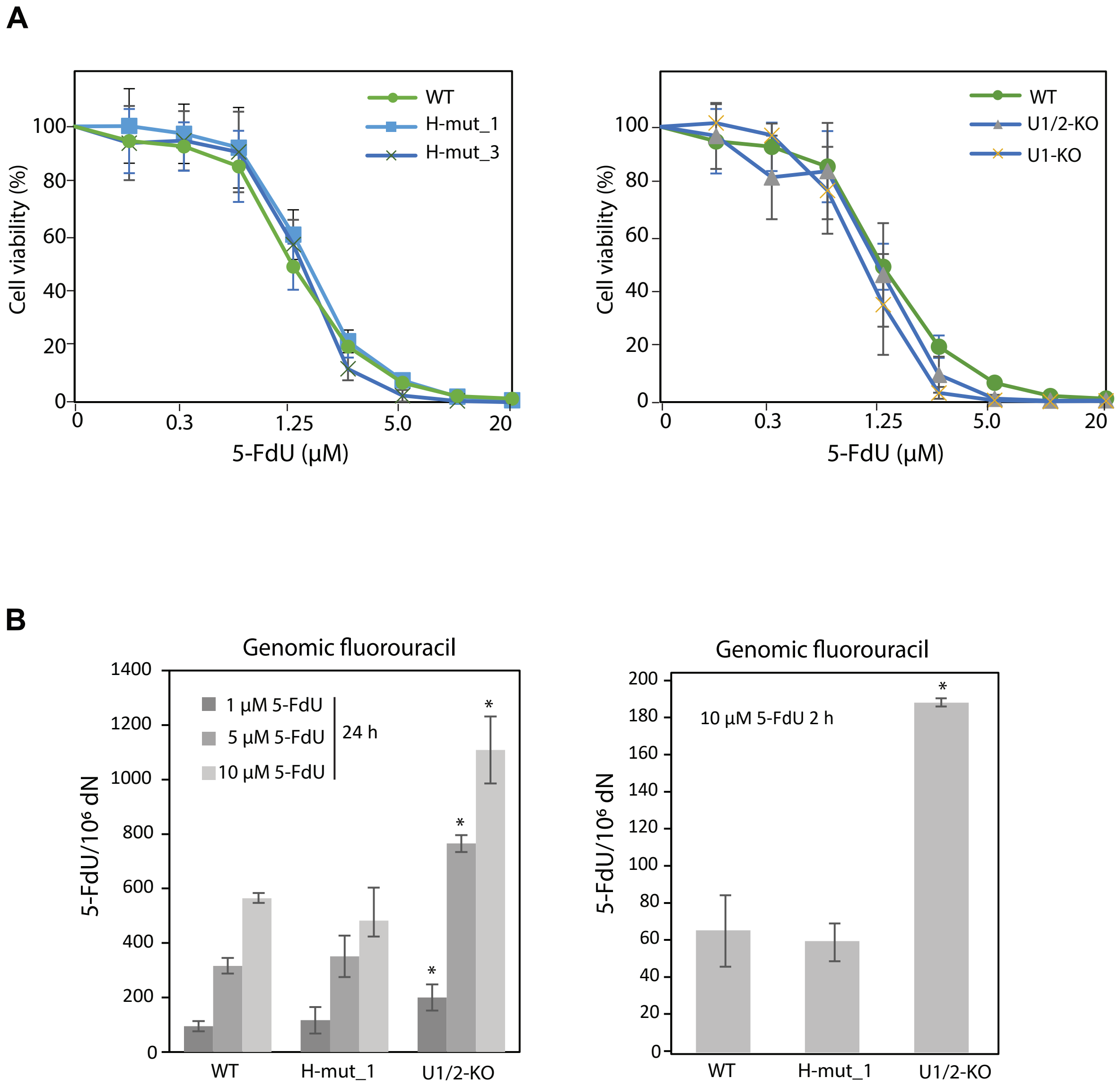

Figure S3. 5-FdU dose-response curves and genomic fluorouracil
A. Cell clones were exposed to the various concentrations of 5-FdU for 72 hrs. Cell viability was measured with Resazurin assay as described. The curves represent the mean of six parallels. All dose-response curves presented in the two panels were from the same experiment.
B. Genomic 5-fluorouracil levels in cell clones measured as 5-fluorodeoxyuridine in hydrolysed genomic DNA isolated from cells treated with increasing concentration of 5-FdU for 24 hours (left panel) or 10 µM 5-FdU for 2 hours (right panel). 5-fluorodeoxyuridine was quantified by LC-MS/MS as described in method section. Bars represent mean of three biological replicates. Asterisk (*) indicates significantly different genomic fluorouracil level compared to similarly treated WT cells (P<0.05, Students T -test, two-tailed, equal variance).

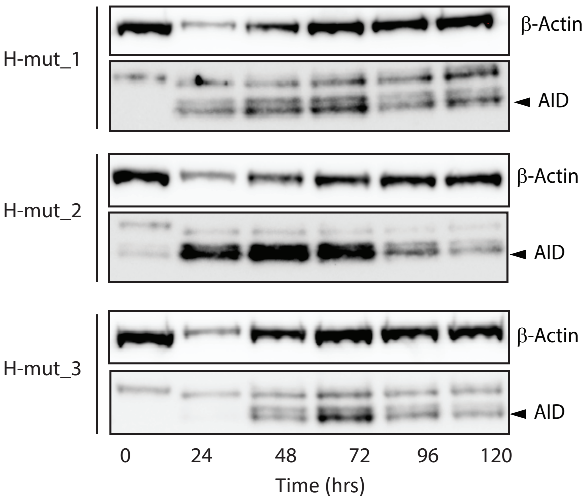

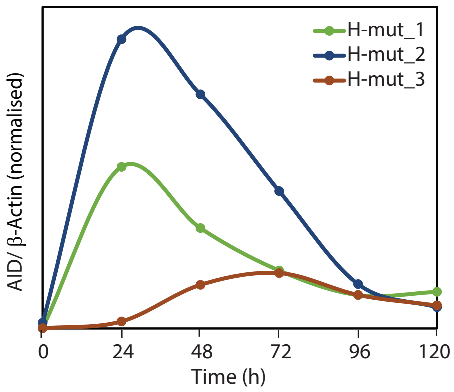

Figure S4. Helix mutants express AID at different levels
Top panels: AID expression after stimulation (+CIT) was compared during a time series of five days (0-120 h) in the three Helix mutants by western blot analysis. AID and β-actin bands were visualised in ChemiDoc^{TM} Imager and quantified by Image Lab software (Bio-Rad). Bottom panels: AID expression (AID/β-Actin) normalised to a WT control loaded on each blot.

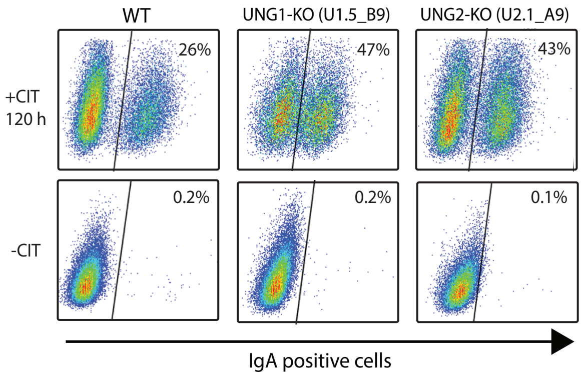

Figure S5. FACS analysis of stimulated (+CIT) and unstimulated (-CIT) CH12F3 clones
IgA switching at harvest (120 h) in additional UNG isotype-specific KO clones prepared for isolation of DNA and mutation analysis. IgA positive (%) cells are indicated in each panel.

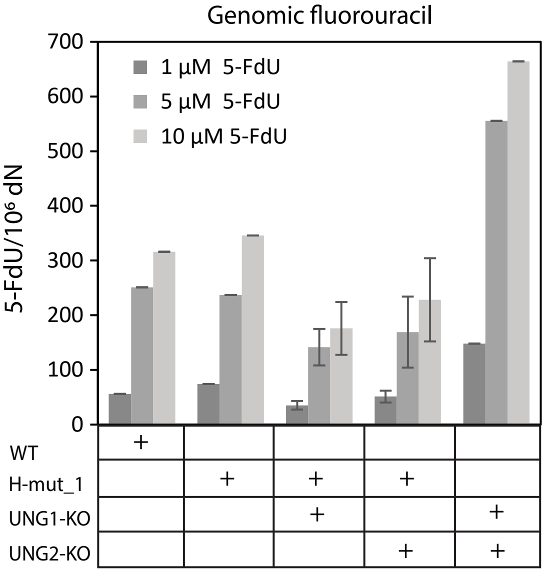

Figure S6. Genomic fluorouracil levels in RPA-binding deficient and isotype specific UNG clones Genomic 5-fluorouracil levels in cell clones measured as 5-fluorodeoxyuridine in hydrolysed genomic DNA isolated from cells treated with increasing concentration of 5-FdU for 24 hours. 5-fluorodeoxyuridine was quantified by LC-MS/MS as described in method section. Bars represent analysed by LC-MS/MS as described in method section. H-mut_1-UNG1-KO; bars represent the mean values of 4 different clones. H-mut_1-UNG2-KO represent the mean of 3 clones. Error bars indicate standard deviation.
